# Subtractive genomics and drug repurposing strategies for targeting *Streptococcus pneumoniae*: insights from molecular docking and dynamics simulations

**DOI:** 10.3389/fmicb.2025.1534659

**Published:** 2025-03-18

**Authors:** Borakha Bura Gohain, Bhaskar Mazumder, Sanchaita Rajkhowa, Sami A. Al-Hussain, Magdi E. A. Zaki

**Affiliations:** ^1^Centre for Biotechnology and Bioinformatics, Dibrugarh University, Dibrugarh, Assam, India; ^2^Department of Pharmaceutical Sciences, Dibrugarh University, Dibrugarh, Assam, India; ^3^Department of Chemistry, Imam Mohammad Ibn Saud Islamic University (IMSIU), Riyadh, Saudi Arabia

**Keywords:** genomic subtraction, *Streptococcus pneumoniae*, molecular dynamics (MD) simulation, drug repurposing, gene ontology

## Abstract

**Introduction:**

*Streptococcus pneumoniae* is a Gram-positive bacterium responsible for severe infections such as meningitis and pneumonia. The increasing prevalence of antibiotic resistance necessitates the identification of new therapeutic targets. This study aimed to discover potential drug targets against *S. pneumoniae* using an *in silico* subtractive genomics approach.

**Methods:**

The *S. pneumoniae* genome was compared to the human genome to identify non-homologous sequences using CD-HIT and BLASTp. Essential genes were identified using the Database of Essential Genes (DEG), with consideration for human gut microflora. Protein-protein interaction analyses were conducted to identify key hub genes, and gene ontology (GO) studies were performed to explore associated pathways. Due to the lack of crystal structure data, a potential target was modeled *in silico* and subjected to structure-based virtual screening.

**Results:**

Approximately 2,000 of the 2,027 proteins from the *S. pneumoniae* genome were identified as non-homologous to humans. The DEG identified 48 essential genes, which was reduced to 21 after considering human gut microflora. Key hub genes included gpi, fba, rpoD, and trpS, associated with 20 pathways. Virtual screening of 2,509 FDA-approved compounds identified Bromfenac as a leading candidate, exhibiting a binding energy of −26.335 ± 29.105 kJ/mol.

**Discussion:**

Bromfenac, particularly when conjugated with AuAgCu_2_O nanoparticles, has demonstrated antibacterial and anti-inflammatory properties against *Staphylococcus aureus*. This suggests that Bromfenac could be repurposed as a potential therapeutic agent against *S. pneumoniae*, pending further experimental validation. The approach highlights the potential for drug repurposing by targeting proteins essential in pathogens but absent in the host.

## Graphical Abstract

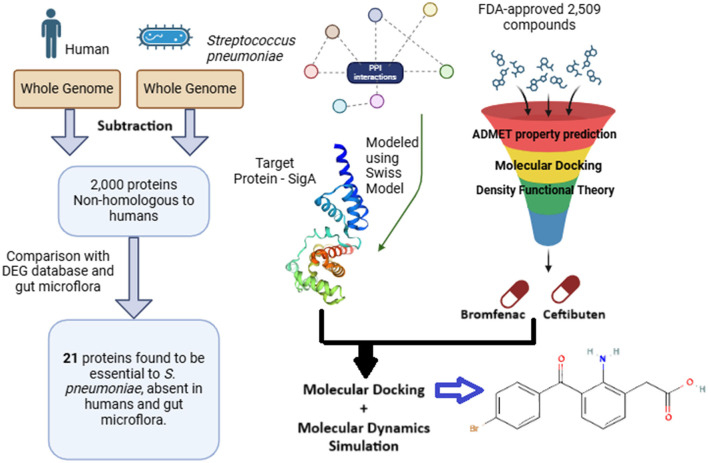

## 1 Introduction

*Streptococcus pneumoniae*, also known as pneumococcus, is a significant global community health concern. This pathogen is the chief cause of: meningitis, bacterial pneumonia, and febrile bacteremia, and is linked to conditions such as otitis media, sinusitis, and bronchitis (Shami et al., 2023). In developing countries, it mainly affects young children and the elderly, resulting in an estimated one million child deaths annually from pneumococcal disease. The WHO emphasizes the urgent need for better vaccines and treatments to combat this pathogen and rising antimicrobial resistance. In 2024, *S. pneumoniae* was added to the WHO's updated Bacterial Priority Pathogens List (BPPL) as a medium-priority pathogen because of its significant disease burden (https://www.who.int/publications/i/item/9789240093461). This inclusion highlights the critical need for enhanced research and development of new therapeutic strategies to address infections caused by this virulent microorganism. Lower respiratory infections resulted in 2.6 million deaths globally in 2013, with a notable increase to 2.74 million in 2015 (McMichael et al., 2006). Beginning in the 1980s, a significant increase in antibiotic intolerance across various regions has been shown by *S. pneumoniae*. Although antibiotics and conjugate vaccines are available, bacterial otitis media remains a leading cause and pre-clinical visits and antibiotic failure are majorly influenced by pneumococcus in the United States. The issue is exacerbated by the prevalence of resistant strains, with resistance to penicillin being displayed by over 40%, which often leads to resistance against other antibiotics such as macrolides and tetracyclines, posing a global health challenge (Musher, 1992). In the United States, the upper respiratory tracts of children are found to contain more than 40% of penicillin-resistant pneumococcal strains (Panwhar and Fiedler, 2018). The growing antibiotic resistance is considered a significant global concern (O'Brien et al., 2009). Additionally, resistance traits and pathogenic factors are capable of being disseminated by *S. pneumoniae* through competence-dependent horizontal gene transfer (McIntosh, 2002). Continuous serotype monitoring and an understanding of the prevalence of drug-resistant strains in the general population are required for effective management of this issue (Sharew et al., 2021).

Invasive Pneumococcal Disease (IPD) is predominantly associated with serotype 14 among the 101 recognized serotypes of *S. pneumoniae* (Geno et al., 2015). The development of conjugated pneumococcal vaccines targeting *S. pneumoniae* infections is based on polysaccharide capsular serotypes (Chiba et al., 2014). For instance, serotype 14 was addressed by the design of the 23-valent Polysaccharide Pneumococcal Vaccine (PPV) for the management of IPD. However, limited immunogenicity against pneumococci has been demonstrated by PPSV23 (Cilloniz et al., 2016). Currently, the Pneumococcal Polysaccharide Vaccine (PPSV23), the 10-valent Pneumococcal Conjugate Vaccine (PCV10), the 7-valent Pneumococcal Conjugate Vaccine (PCV7), and the 13-valent Pneumococcal Conjugate Vaccine (PCV13) are in use. Despite the introduction of multi-valent PCV7, a noted increase in serotype 14 infections over time has been observed, which has been attributed to the rise in drug resistance (Al-Jumaili et al., 2023).

The gold standard methods used to study outbreaks and identify pneumococcal isolates, such as MultiLocus Sequence Typing (MLST) and Pulse-Field Gel Electrophoresis (PFGE), are molecular serotyping (Enright and Spratt, 1998), but high associated costs are encountered. Thus, a challenge has been presented by the accurate determination of the serotypes (Hu et al., 2014). It is therefore imperative that a new novel therapeutic drug target against *S. pneumoniae* is identified (Khan et al., 2022). Better therapeutics may be led to by the discovery of a new drug target (Lodha et al., 2013). Fortunately, new strategies have been introduced through advancements in the post-genomic era and whole-genome sequencing of pathogens, including comparative subtractive genomics, for developing novel drugs and vaccine candidates. Additionally, potential drug targets against these pathogens can be identified using computational approaches (Fair and Tor, 2014).

The subtractive genomic approach is used to compare host and pathogen genomes to identify essential pathogen-specific proteins that are absent in the host (Barh et al., 2011; Bottacini et al., 2014; Uddin et al., 2015, 2020; Uddin and Saeed, 2014). Genes critical for pathogen survival, replication, and sustainability are highlighted, enabling the identification of therapeutic targets that do not affect host biology. By focusing on non-host genes involved in distinct metabolic pathways, pathogen function can be disrupted while minimizing potential side effects (Uddin et al., 2020; Wadood et al., 2018). Computational studies are utilized to prioritize target genes, streamline experimental efforts, and reduce the need for extensive research. The integration of multi-omics data with structural and functional analysis is employed to refine target selection, ensuring a systematic approach that filters out paralogous and homologous sequences while focusing on non-paralogous sequences essential for pathogen viability. Overall, subtractive genomics is recognized as a valuable tool for identifying promising therapeutic targets and facilitating efficient drug development.

The current strategy for combating resistant pathogens focuses on identifying unique and innovative drug targets within the bacterial genome. Various methodologies, particularly computational subtractive genomics analysis, are employed to pinpoint these new drug targets effectively (Barh et al., 2011; Uddin et al., 2015; Wadood et al., 2018). In this research, a computational subtractive genomics approach was utilized to discover novel targets against *S. pneumoniae*. This involved high-throughput screenings of the *S. pneumoniae* genome and human gut bacteria genome against the human genome to identify non-homologous sequences using CD-HIT. The Database of Essential Genes (DEG) was integrated into the analysis to detect potential drug targets, alongside differential pathway analysis and subcellular localization assessments. This process revealed druggable, non-homologous essential proteins of *S. pneumoniae*, providing valuable insights through GO and metabolic pathway evaluations (Ali et al., 2023). Additionally, a drug repurposing approach consisting of structure-based virtual screening was applied to potentially inhibit the target protein.

## 2 Materials and methods

**Figure 1:**
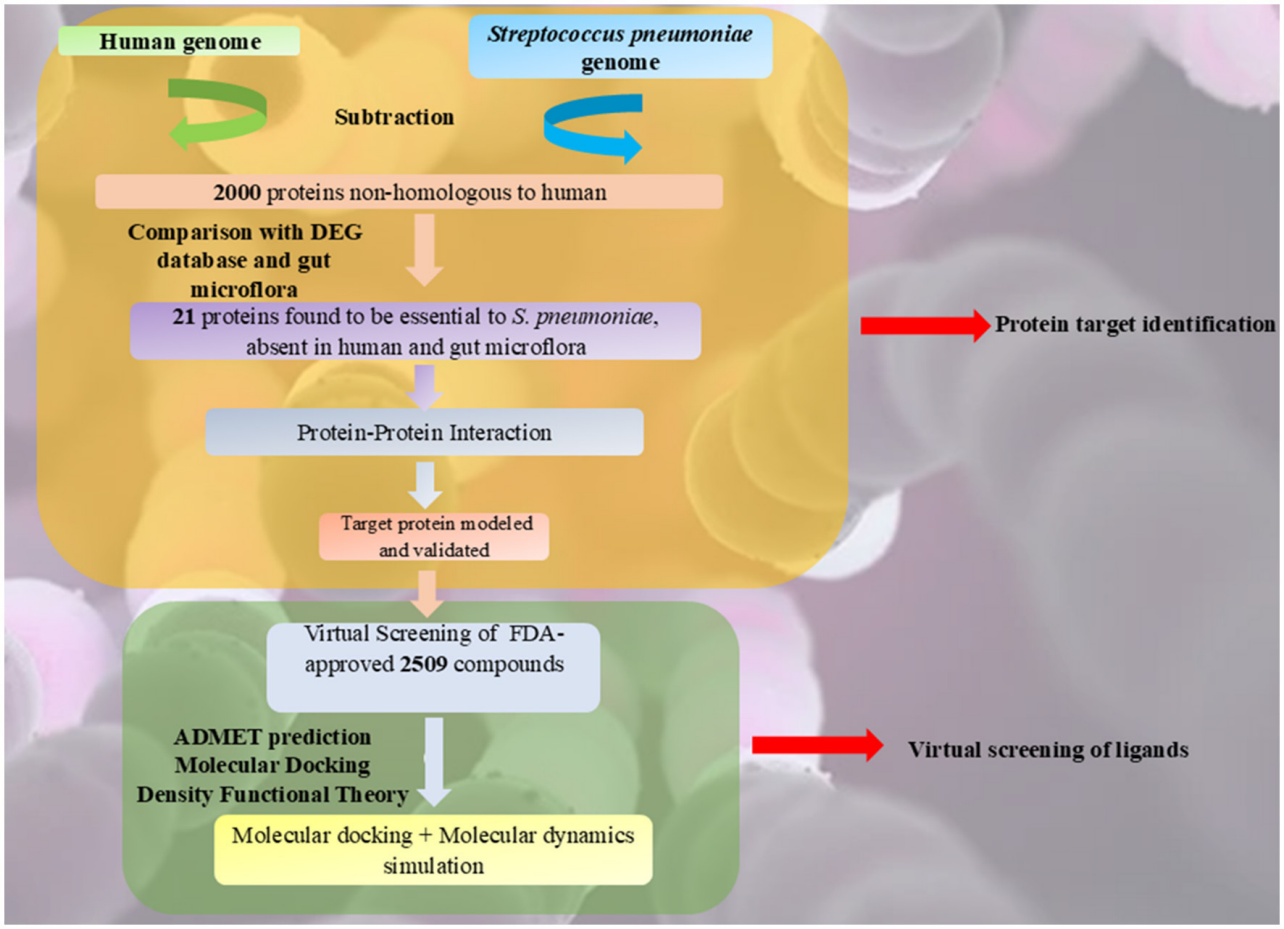
Workflow of the study.

The current methodology consists of two stages, as illustrated in Figure 1. In the first stage, distinctive and potentially druggable targets in *S. pneumoniae* were identified using a subtractive genomics approach, which involved the analysis of metabolic pathways and gene ontology (GO), followed by homology modeling of the target protein. This approach has been effectively used to prioritize potential drug targets (Khan et al., 2022). Various databases and computational tools were utilized to identify therapeutic targets against *S. pneumoniae*.

In the second stage, virtual screening of 2,509 FDA-approved compounds was performed using ADMET prediction, molecular docking, and Density Functional Theory (DFT) to identify potential repurposed inhibitors. Molecular docking and molecular dynamics simulations were then conducted to determine the most potent repurposed inhibitor.

### 2.1 Obtaining genomes of both the bacteria and the host organism

The complete genome assemblies of *S. pneumoniae* (GCF_002076835.1_ASM207683v1_protein.fasta) and humans (GCF_000001405.40_GRCh38.p14_protein.fasta) were accessed from the National Center for Biotechnology Information (NCBI; Sayers et al., 2022) website (https://www.ncbi.nlm.nih.gov/). Essential protein sequences for prokaryotic organisms were retrieved from DEG (Zhang, 2004; http://origin.tubic.org/deg/public/index.php).

### 2.2 Elimination of duplicate sequences

The genome of *S. pneumoniae* was processed using CD-HIT (version 4.8.1) with a 90% identity threshold (Fatoba et al., 2021; Fu et al., 2012). This tool, which is commonly used for clustering and comparing protein and genomic sequences, was employed to remove redundant or duplicate proteins (Huang et al., 2010). As a result, duplicate protein sequences were filtered out, leaving only the unique sequences for subsequent analysis.

### 2.3 Recognition of non-similar proteins and assessment of gut microbiota

Protein sequences in *S. pneumoniae* lacking homologs in human proteins were identified using a BLASTp search against the *Homo sapiens* genome (Fatoba et al., 2021), with an *E*-value cut-off of 10^−5^. Sequences with notable similarity to human proteins were excluded, while non-homologous sequences were retained for further analysis.

Since the gut microbiota plays a crucial role in maintaining health and influencing disease states, interactions between humans and their gut microorganisms are predominantly mutualistic and symbiotic rather than merely commensal (Savage, 1977; Sears, 2005). These microorganisms offer numerous benefits, such as preventing pathogen proliferation, fermenting inactive energy substrates, modulating immune responses, regulating gastrointestinal growth, synthesizing essential vitamins (e.g., vitamin K and biotin), producing fat storage-related hormones, and providing disease protection (Guarner and Malagelada, 2003). However, unintended inhibition of key microbial proteins could be detrimental. To assess this, the selected non-human *S. pneumoniae* proteins were compared with the genomes of human gut microorganisms, obtained from various literature sources (Anis Ahamed et al., 2021) and the mBodyMap Database (Jin et al., 2022), using a BLASTp search with an *E*-value cut-off of 10^−5^. Additionally, a BLASTp analysis of *S. pneumoniae* non-homologous proteins were performed against the DEG database, identifying essential proteins with an E-value threshold of 10^−100^.

### 2.4 Recognition of vital non-similar genes

Proteins crucial to cellular metabolism are present in all organisms (Deng et al., 2011). Therefore, a BLASTp analysis was performed on the non-homologous proteins of *S. pneumoniae* against the DEG database. Proteins deemed essential in *S. pneumoniae* were identified by applying a stringent *E*-value threshold of 10^−100^. A minimum cut-off score of 100 was used to select essential genes (Fatoba et al., 2021). This approach yielded a dataset of proteins that are both non-homologous to humans and essential for *S. pneumoniae*.

### 2.5 Analysis of UniProt ID mapping and evaluation of drug potential in selected sequences

The UniProt ID Mapper facilitates the conversion of protein identifiers across biological databases (Zaru et al., 2023), providing a centralized platform for data integration and standardization. By automating this process, it efficiently links identifiers from diverse databases, enhancing interoperability, and supporting bioinformatics analyses. To identify potential new drug targets, all critical, unique, and predicted protein sequences were cross-referenced with the DrugBank database (Knox et al., 2011), which contains targets for FDA-approved drug molecules.

### 2.6 Analysis of sequence alignment using the EMBOSS needle tool for pairwise comparisons

Pairwise sequence alignment analysis was conducted using EMBOSS Needle to compare two biological sequences for the identification of regions showing similarity or homology (Ionescu, 2019; Panwar et al., 2015). This tool, which is part of the EMBOSS suite (Rice et al., 2000), employs the Needleman-Wunsch algorithm for aligning sequences, facilitating the exploration of evolutionary relationships, functional similarities, and structural motifs.

### 2.7 Subcellular localization identification

Proteins are classified into various subcellular regions, such as the cytoplasm, inner membrane, periplasmic space, and outer membrane, using localization prediction methods. Potential drug and vaccine targets are identified among proteins located in the cytoplasm and outer membrane, respectively. Accurate localization is deemed essential for understanding protein function and interactions, thereby aiding the development of targeted therapies. The function of specific proteins is regarded as critical for identifying therapeutic targets, as proper subcellular localization is necessary for protein activity. UniProt was employed for this analysis, and the results were validated using the CELLO v.2.5 online tool (Shami et al., 2023). It has been demonstrated that, due to the ability of proteins to localize in multiple cellular compartments, understanding their localization is vital for the design of effective therapeutic strategies.

### 2.8 Exploration of protein connectivity in networks

Protein-protein interaction (PPI) networks for the proteins were sourced from STRING database version 12.0 (https://string-db.org/; Szklarczyk et al., 2011). PPIs are fundamental to cellular signaling and transduction, marking them attractive therapeutic drug development targets (Nada et al., 2024). Recent technological advances have made targeting these interactions increasingly feasible. These networks were constructed and visualized using Cytoscape 3.7.2 (Shannon et al., 2003). After merging the networks of the targets to illustrate the interactions among all selected proteins, a topological analysis was conducted. The central node within the network was identified using the cytohubba plugin (Chin et al., 2014).

### 2.9 Gene ontology analysis and pathway analysis using ShinyGo

Functional enrichment of gene lists was assessed using ShinyGO 0.80 (Ge et al., 2020; Hannan et al., 2024). Enrichment was evaluated across three GO categories: biological processes (BP), molecular functions (MF), and cellular components (CC). Pathway analysis was also performed to identify significantly enriched pathways using resources such as KEGG (Kyoto Encyclopedia of Genes and Genomes) and Reactome. Default parameters were applied for the analyses, and results were visualized through bar charts and dot plots, which displayed relevant GO terms and pathways along with their *p*-values and enrichment scores. Insights into the biological functions and interactions of the gene sets were provided by this combined approach.

### 2.10 Protein framework development and assessment

Due to the lack of an experimentally determined crystal structure for rpoD, the 3D model of the protein was created using homology modeling. The rpoD sequence from *S. pneumoniae* was sourced from UniProt (https://www.uniprot.org/) with the ID WP_000201898 and was used to build the model via Swiss Model (Schwede, 2003). Refinement of the initial models was performed with the Galaxy web server (Ko et al., 2012). The models' quality was evaluated through tools such as Verify3D, ERRAT, and Procheck (https://saves.mbi.ucla.edu/; Shami et al., 2023), and secondary structure predictions were made using PSIPRED (McGuffin et al., 2000). Stability and conformational dynamics of the model were examined with molecular dynamics (MD) simulations over a 100 ns timeframe (Khataniar et al., 2023).

### 2.11 Binding site prediction

Structural pockets and cavities are often associated with the binding and active sites of proteins. In this study, the binding site of the modeled protein was predicted using UniProt (https://www.uniprot.org/) and the Motif search tool (https://www.genome.jp/tools/motif/), along with the FTMap server (Das et al., 2024; Kozakov et al., 2015) and the CASTp server (http://sts.bioe.uic.edu/castp/; Tian et al., 2018). Additionally, a comprehensive review of the relevant literature was conducted. The identified active sites were utilized for Molecular docking studies with ligands and the respective protein targets.

### 2.12 Virtual screening of FDA-approved compounds

#### 2.12.1 ADMET

A collection of 2,509 FDA-approved drugs was obtained from the DrugBank database, and ADMET screening was carried out using Discovery Studio to filter out undesirable ligands. This screening included evaluations of parameters such as aqueous solubility, blood-brain barrier permeability, CYP2D6 binding, hepatotoxicity, intestinal absorption, and plasma protein binding. Further toxicity predictions were made using the Ames mutagenicity model to exclude unsuitable ligands.

#### 2.12.2 Molecular docking

The protein was refined based on the subtraction genomics study, and molecular docking of the ligands that qualified the ADMET were docked with the target using the LibDock module (Tai et al., 2023) within Discovery Studio. LibDock is extensively used for the virtual screening of compound libraries to identify potential drug candidates. This high-throughput docking approach facilitates the rapid screening of large chemical libraries by assessing ligand binding poses and interactions within a target protein's active site.

#### 2.12.3 Density Functional Theory

Properties such as electron affinities, ionization potentials, orbital energies, and molecular structures were assessed through DFT analysis (Rajkhowa et al., 2022). This analysis focused on HOMO-LUMO frontier orbitals, which indicate the chemical reactivity of the compounds. A higher E_HOMO_ value indicates a greater tendency for a molecule to donate electrons, while the E_LUMO_ value reflects its electron-accepting ability. A smaller HOMO-LUMO gap (δE) was associated with increased molecular reactivity and decreased stability of the compound. These calculations were executed in Discovery Studio using DMol^3^ with the B3LYP functional and the DNP basis set. Ligands were selected based on energy gap, ensuring optimal stability and binding affinity for the target protein.

### 2.13 Protein-drug complexes: molecular dynamics simulations

MD simulations were carried out to evaluate the stability and conformational changes of the proposed model, as well as to examine protein-ligand interactions over various time frames (Rajkhowa et al., 2017). The model protein underwent MD simulation for 100 ns, while the ligand-protein complex was simulated for 50 ns using the GROMACS package version 2021.4 with the GROMOS54a7 force field. The protein was placed within a cubic periodic box and was solvated using the SPC water model, with a separation of ~1.0 nm between the solute molecules and the boundaries of the box. Energy minimization was followed by a 50 ns equilibration phase at a pressure of 1 bar and a temperature of 298 K, employing Berendsen coupling. The production dynamics simulation was subsequently conducted in an NVT ensemble at 298 K.

The RMSD (root mean square deviation) was calculated to determine the average positional deviation between atom groups in the protein-ligand complex relative to the protein frame, providing insights into complex stability. The RMSF (root mean square fluctuation) was used to analyze the average deviation of individual residues from their reference positions, highlighting regions of the protein with the greatest variability compared to the reference structure. To assess the compactness of the protein structure, the radius of gyration (Rg) was measured, which indicates the distance of protein residues from the center of mass, thus providing insights into the overall compactness and folding state of the protein structure (Gl et al., 2020). Using the Automated Topology Builder (version 3.0) online tool (https://atb.uq.edu.au/; Stroet et al., 2018), the topology of selected ligands was generated (Saha and Jha, 2024).

### 2.14 Calculations of binding-free energy

The binding free energy was assessed through the Molecular Mechanics Poisson-Boltzmann Surface Area (MM-PBSA) approach within GROMACS (Manhas et al., 2019; Rajkhowa et al., 2022). This technique combines molecular mechanics energy with solvation energy derived from the Poisson-Boltzmann equation and the solvent-accessible surface area (SASA).

Molecular mechanics energies, including bond, angle, torsional, and non-bonded interactions, were computed from MD trajectory. Solvation-free energies were estimated using the Poisson-Boltzmann equation, with SASA contributions for non-polar interactions.

MM-PBSA were chosen for its efficiency and accuracy in evaluating binding energies. It effectively analyzes protein-ligand, protein-protein interactions, and conformational changes, providing insights into the energetic contributions and forces driving molecular recognition and stability.

## 3 Results

The study was conducted to identify novel drug targets in *S. pneumoniae*, a bacterium responsible for severe infections such as meningitis, bacteremia, and pneumonia. In developing nations, it is associated with acute lower respiratory tract infections, causing ~5 million deaths annually among children under five (Sheoran et al., 2022). Increasing penicillin resistance and limited vaccine efficacy have led to rising morbidity and mortality, while drug development remains slow due to high costs and the requirement for specialized expertise, further complicated by emerging drug-resistant strains.

Advancements in bioinformatics have facilitated drug discovery, with subtractive genomics widely utilized to identify pathogen-specific targets through *in silico* proteome analysis. This approach allows for the selection of essential bacterial proteins without affecting the host genome, thereby minimizing toxicity. In this study, subtractive genomics was employed to identify potential drug targets in *S. pneumoniae*, a method previously applied to characterize unique targets in human pathogens (Wadhwani and Khanna, 2016).

### 3.1 Paralogous protein sequence elimination

The complete proteome of *S. pneumoniae*, comprising 2027 protein sequences, was retrieved from the NCBI database in FASTA format. The aim of the study was to identify unique, essential proteins specific to the pathogen that could be potential therapeutic targets. After retrieving the proteome, paralogous sequences were removed to improve the precision of subsequent analyses. This task was performed using the CD-HIT tool, which reduced the proteome to 2016 proteins by eliminating 11 redundant sequences.

### 3.2 Profiling of non-homologous proteins

Similarity between the pathogen's proteins and those of the host may be observed. Therefore, it is necessary to identify and exclude these homologous host protein sequences from the pathogen's proteome to mitigate potential toxicity to host cells. This was accomplished by using BLASTp with an *E*-value cutoff of 10^−5^. Following the BLASTp analysis, 2000 non-homologous sequences were identified.

### 3.3 Identification of crucial genes that are non-homologous

The development and growth of pathogens are significantly influenced by essential proteins. These proteins are deemed highly promising and secure targets for drug development. DEG was employed to pinpoint these crucial proteins, leading to the identification of 48 essential proteins vital for the pathogen's survival.

### 3.4 Screening of human gut metagenomes

Antibiotics, which often impact both pathogenic and beneficial bacteria in the human microbiota, can lead to prolonged disruption of normal gut flora (Willing et al., 2011). To reduce the risk of broadside effects, pathogen proteins similar to gut flora proteins were identified and excluded as potential drug targets. The complete genome of *S. pneumoniae* was used as a query against reference genomes of gastrointestinal flora using BLASTp within the mBodyMap Database. Of 2,087 gut bacterial sequences, 87 were found to be homologous to gut flora, while 2,000 sequences were classified as non-homologous. A BLASTp search against the human genome revealed 1,981 proteins with similarities to human proteins, leaving 27 proteins (with an *E*-value of 10^−5^) as dissimilar to the human genome. Additionally, a BLASTp search against DEG identified 21 essential genes for the survival of *S. pneumoniae* (with an *E*-value of 10^−100^), indicating their potential as effective drug targets.

### 3.5 UniProt ID mapper analysis

The UniProt ID mapper was used to analyze the 21 essential proteins, resulting in 168 mapped entries: eight were reviewed (Swiss-Prot) and 160 were unreviewed (TrEMBL). This mapping revealed that the reviewed proteins were associated with two *S. pneumoniae* strains: serotype 4 (ATCC BAA-334/TIGR4; Williams et al., 2012) and strain ATCC BAA-255/R6. Key proteins identified included RNA polymerase sigma factor SigA, oligopeptide transport system permease protein AmiD, fructose-bisphosphate aldolase, and tryptophan—tRNA ligase. These proteins were found in both *S. pneumoniae* serotype 4 and strain R6, indicating their conservation across these strains. Valuable insights into the molecular biology and potential therapeutic targets of this important human pathogen are provided by the detailed data from the ID mapping resource, as presented in Table 1.

**Table 1:** UniProt ID mapper result.

**Sl_no**.	**From**	**Entry**	**Protein names**	**Gene names**	**Organism**	**Length**
1	WP_000201898.1	POA4I9	RNA polymerase sigma factor SigA	sigA, rpoD, SP_1073	*S. pneumoniae* serotype 4 (strain ATCC BAA-334/TIGR4)	369 AA
2	WP_000201898.1	POA4JO	RNA polymerase sigma factor SigA	sigA, rpoD, spr0979	*S. pneumoniae* (strain ATCC BAA-255/R6)	369 AA
3	WP_000103700.1	POA4M9	Oligopeptide transport system permease protein AmiD	amiD, SP_1889	*S. pneumoniae* serotype 4 (strain ATCC BAA-334/TIGR4)	308 AA
4	WP_000103700.1	POA4NO	Oligopeptide transport system permease protein AmiD	amiD, spr1705	*S. pneumoniae* (strain ATCC BAA-255/R6)	308 AA
5	WP_001019003.1	POA4S1	Fructose-bisphosphate aldolase	fba, SP_0605	*S. pneumoniae* serotype 4 (strain ATCC BAA-334/TIGR4)	293 AA
6	WP_001019003.1	POA4S2	Fructose-bisphosphate aldolase	fba, spr0530	*S. pneumoniae* (strain ATCC BAA-255/R6)	293 AA
7	WP_000165444.1	P67595	Tryptophan—tRNA ligase	trpS, SP_2229	*S. pneumoniae* serotype 4 (strain ATCC BAA-334/TIGR4)	341 AA
8	WP_000165444.1	P67596	Tryptophan—tRNA ligase	trpS, spr2034	*S. pneumoniae* (strain ATCC BAA-255/R6)	341 AA

### 3.6 Potential for drug development in selected sequences

The potential for drug development targeting essential proteins in *S. pneumoniae* was assessed, leading to the identification of eight proteins that correspond to targets of FDA-approved drugs as listed in the DrugBank database (Table 2). Based on literature evidence (Williams et al., 2012) indicating the avirulence of strain ATCC BAA-255/R6, **serotype 4 (strain ATCC BAA-334/TIGR4)** was selected, and four proteins, including the **RNA polymerase sigma factor SigA**, **Oligopeptide transport system permease protein AmiD**, **Fructose-bisphosphate aldolase**, and **Tryptophan—tRNA ligase**, were chosen for further investigation.

**Table 2:** Findings from comparative sequence alignment evaluation.

**Sl_no**.	**Protein names**	**Gene names**	***S. pneumoniae***	**Name of the organism in which reported (from DrugBank)**	**Identity (in %)**	**Sub-cellular localization UniProt and CELLO v.2.5**
1	RNA polymerase sigma factor SigA	sigA, rpoD, SP_1073	*S. pneumoniae serotype* 4 (strain ATCC BAA-334/TIGR4)	*Clostridioides difficile* (strain 630)	56.7	Cytoplasmic
2	RNA polymerase sigma factor SigA	sigA, rpoD, spr0979	*S. pneumoniae* (strain ATCC BAA-255/R6)	*Thermus thermophilus* (strain HB8/ATCC 27634/DSM 579)	44	Cytoplasmic
3	Oligopeptide transport system permease protein AmiD	amiD, SP_1889	*S. pneumoniae serotype* 4 (strain ATCC BAA-334/TIGR4)	–	–	Cell membrane multi-pass membrane protein
4	Oligopeptide transport system permease protein AmiD	amiD, spr1705	*S. pneumoniae* (strain ATCC BAA-255/R6)	–	–	Cell membrane multi-pass membrane protein
5	Fructose-bisphosphate aldolase	fba, SP_0605	*S. pneumoniae serotype* 4 (strain ATCC BAA-334/TIGR4)	*Plasmodium falciparum*	7.7	Cytoplasmic
6	Fructose-bisphosphate aldolase	fba, spr0530	*S. pneumoniae* (strain ATCC BAA-255/R6)	–	–	Cytoplasmic
7	Tryptophan—tRNA ligase	trpS, SP_2229	*S. pneumoniae serotype* 4 (strain ATCC BAA-334/TIGR4)	*Geobacillus stearothermophilus*	33.6	Cytoplasmic
8	Tryptophan—tRNA ligase	trpS, spr2034	*S. pneumoniae* (strain ATCC BAA-255/R6)	–	–	Cytoplasmic

To identify and evaluate druggable proteins in *S. pneumoniae*, the DrugBank database was utilized. Four unique, essential, and non-homologous proteins were examined, with three proteins associated with serotype 4 (strain ATCC BAA-334/TIGR4) and one associated with strain ATCC BAA-255/R6. Table 2 provides detailed information on drug targets identified, highlighting three proteins with potential as drug targets. Of these, three proteins are localized in the cytoplasmic region, while one is associated with the cell membrane. Cytoplasmic proteins are often considered favorable therapeutic targets (Khan et al., 2022).

### 3.7 Pairwise sequence alignment analysis

The sequence identity between *S. pneumoniae* proteins and their homologs in other organisms (such as *Clostridioides difficile, Thermus thermophilus, Plasmodium falciparum*, and *Geobacillus stearothermophilus*) represents the percentage of identical amino acids in aligned regions of their protein sequences. In this study, sequence identity values ranged from 7.76% to 56.7% (Table 2), reflecting varying evolutionary and functional diversity among the proteins. Higher identity values indicate conserved regions crucial for protein function, while lower values suggest divergence and potential functional differences. Despite their presence in other organisms, the low sequence identity makes these proteins suitable candidates for further investigation.

The identified target was re-evaluated applying the BRENDA enzyme database (https://www.brenda-enzymes.org; Chang et al., 2021). Among the three targets of interest, Fructose-bisphosphate aldolase was found in *Homo sapiens*. The results showed a sequence identity of only 14.4% (Table 3). This confirms that these targets are viable for drug development. Additionally, the low sequence identity implies minimal cross-reactivity, reducing the risk of off-target effects. Further studies will explore the therapeutic potential of these targets.

**Table 3:** Summary of sequence similarity.

**Sl_no**.	**Protein names**	**Organisms (Brenda and UniProt)**		**Seq identity**	**Seq similarity**
		***S. pneumoniae***	***Homo sapiens***		
1	RNA polymerase sigma factor SigA	POA4I9	No	–	–
2	Fructose-bisphosphate aldolase	POA4S1	P05062, P04075	14.4%	22.7%
3	Tryptophan–tRNA ligase	P67595	No	–	–

The RNA polymerase sigma factor SigA in *S. pneumoniae* was selected due to its critical role in bacterial transcription regulation. Unlike fructose-bisphosphate aldolase, which shares 22.7% sequence similarity with its human homolog, SigA and tryptophan-tRNA ligase (TrpS) do not exhibit such similarity. Transcription initiation is critically dependent on SigA, which directs RNA polymerase to specific promoter sequences and facilitates the transcription of housekeeping genes essential for cellular growth and maintenance (Kazmierczak et al., 2005; Paget, 2015). Insights into SigA's structure and function are crucial for developing targeted antibacterial therapies (Feklístov et al., 2014).

Although TrpS plays a vital role in protein synthesis by charging tRNA with tryptophan, its impact is narrower compared to SigA. The focus on SigA underscores the importance of targeting bacterial transcription mechanisms. Expression of various crucial genes can be impaired by the inhibition of SigA, potentially resulting in more effective antibacterial strategies (Murakami and Darst, 2003).

The primary sigma factor, σ70 (SigA), is encoded by the rpoD gene in bacteria. Essential for initiating transcription, SigA binds to RNA polymerase (RNAP) and recognizes promoter sequences (Große et al., 2022; Miura et al., 2015). The transcription of housekeeping genes necessary for bacterial growth and survival is facilitated by SigA. Additionally, the expression of horizontally acquired genes, including those related to antibiotic resistance and virulence, is regulated by SigA. Multiple critical genes can be disrupted simultaneously by targeting SigA, making it a promising target for broad-spectrum antibacterial strategies. In contrast, trpS is involved in tryptophan biosynthesis (Martins et al., 2024), which is a more specific function. Therefore, the importance of targeting bacterial transcription mechanisms is highlighted by focusing on rpoD (SigA), as interfering with SigA can impact a wide range of essential genes, making it a more significant target compared to trpS.

### 3.8 Structure of protein-protein interaction networks

**Figure 2:**
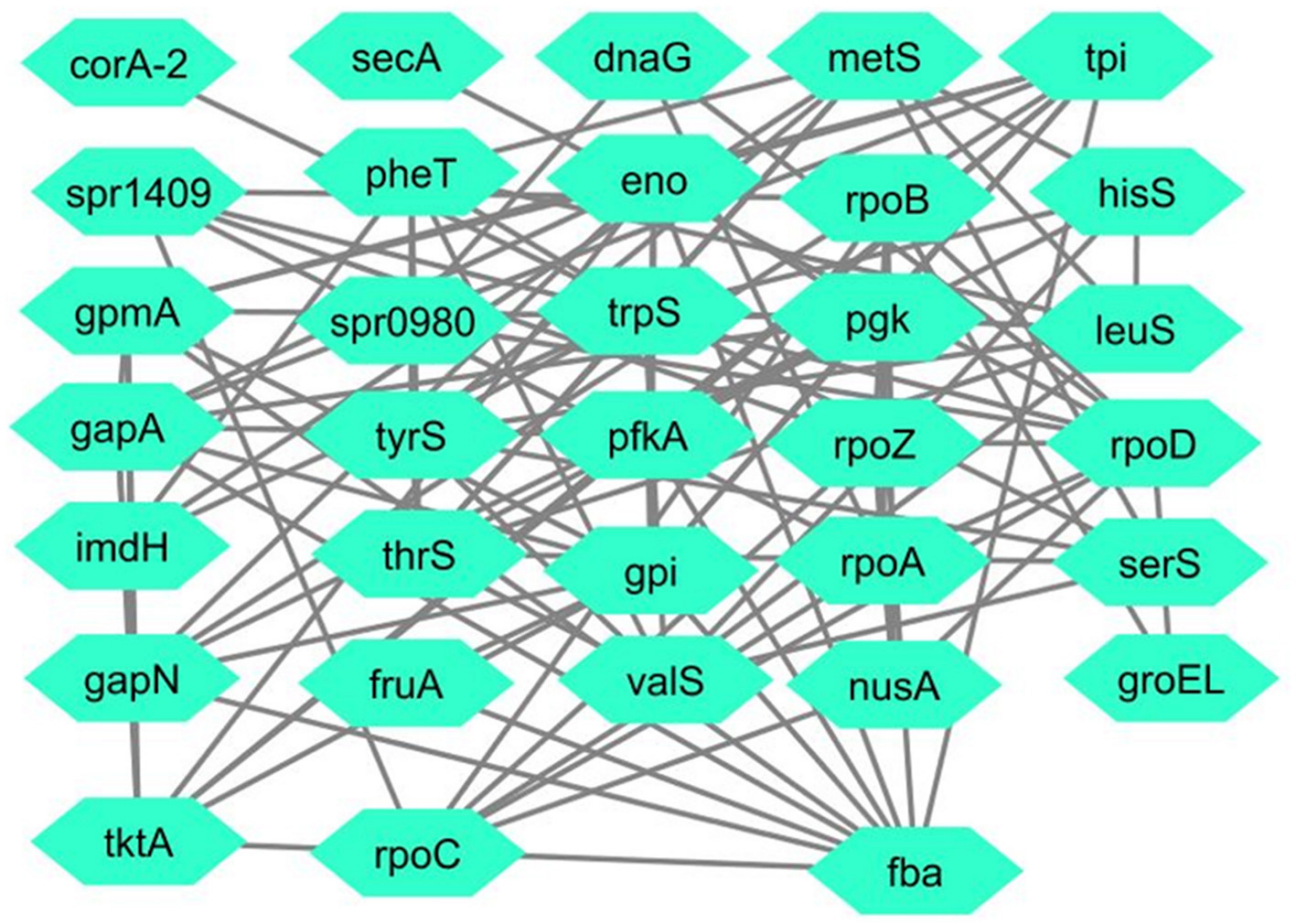
Merged PPI of RNA polymerase sigma factor SigA, fructose-bisphosphate aldolase, and tryptophan–tRNA ligase.

Interactions among proteins (Schwartz et al., 2009), including RNA polymerase sigma factor SigA, fructose-bisphosphate aldolase, and tryptophan-tRNA ligase, were obtained from the STRING database with a confidence score of 0.007. These interactions were used to construct a protein-protein interaction network, as illustrated in Figure 2. The network was analyzed to examine the relationships between the proteins. The oligopeptide transport system permease protein AmiD was not included in the STRING database, so it was omitted from the analysis, leaving the remaining three proteins for further study.

### 3.9 Assessment of crucial genes

**Figure 3:**
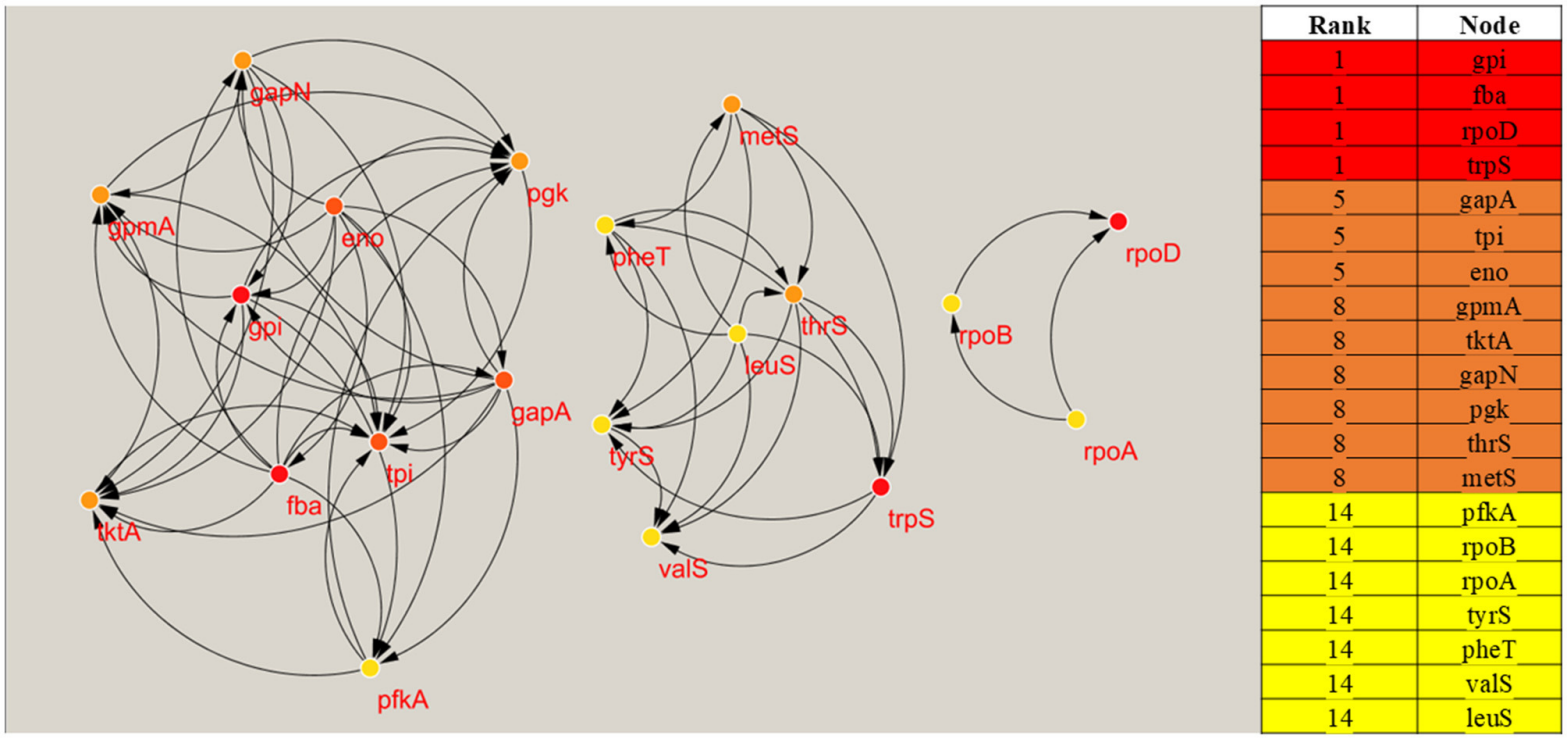
Displaying the significant hub genes along with their rank scores, with red indicating the most important hub genes, orange signifying moderate importance, dark orange representing average importance, and yellow denoting the lowest importance.

The Protein-Protein Interaction network was displayed and examined with Cytoscape (Figure 3). To pinpoint hub genes, or proteins with the highest connectivity in the network, the CytoHubba plugin of Cytoscape version 3.7.2 was utilized. These hub genes were considered critical components of the *S. pneumoniae* genome, indicating their potential as targets for selective antibacterial therapies. As illustrated in Figure 3, the top 20 core targets identified were gpi, fba, rpoD, trpS, gapA, tpi, eno, gpmA, tktA, gapN, pgk, thrS, metS, pfKA, rpoB, rpoA, tyrS, and pheT. Four genes—gpi, fba, rpoD, and trpS—were identified as key hub genes due to their high connectivity, which is determined by the number of nodes associated with each protein. Of these four hub genes, **fba, rpoD**, and **trpS** were selected for further investigation due to their significant involvement in relevant pathways, whereas **gpi** was excluded, as it was not detected in the UniProt ID analysis.

### 3.10 Gene ontology evaluation and pathway exploration

The GO evaluation was performed with ShinyGO version 0.80 (Ge et al., 2020; Ramesh Babu, 2023) revealed significant insights into gene pathways associated with the studied genes. Various sorting criteria were employed to identify the most relevant pathways, including fold enrichment, false discovery rate (FDR), the average of FDR and fold enrichment, the number of genes, and a combined metric of FDR and fold enrichment. Notably, pathways were identified by sorting with Avg_rank (FDR and fold enrichment), resulting in a total of 211 pathways, indicating a comprehensive range of BP, MF, and CC. In contrast, sorting by rpoD yielded only 41 pathways, highlighting a more focused selection based on specific gene associations as shown in Supplementary Table 2.

These results underscore the effectiveness of ShinyGO in GO analysis, as significant biological insights were derived through customizable sorting and filtering capabilities tailored to the research objectives. The findings also illustrate the potential for ShinyGO to enhance data visualization and facilitate integration with other bioinformatics tools, thereby establishing it as a valuable resource for diverse genomic studies.

**Figure 4:**
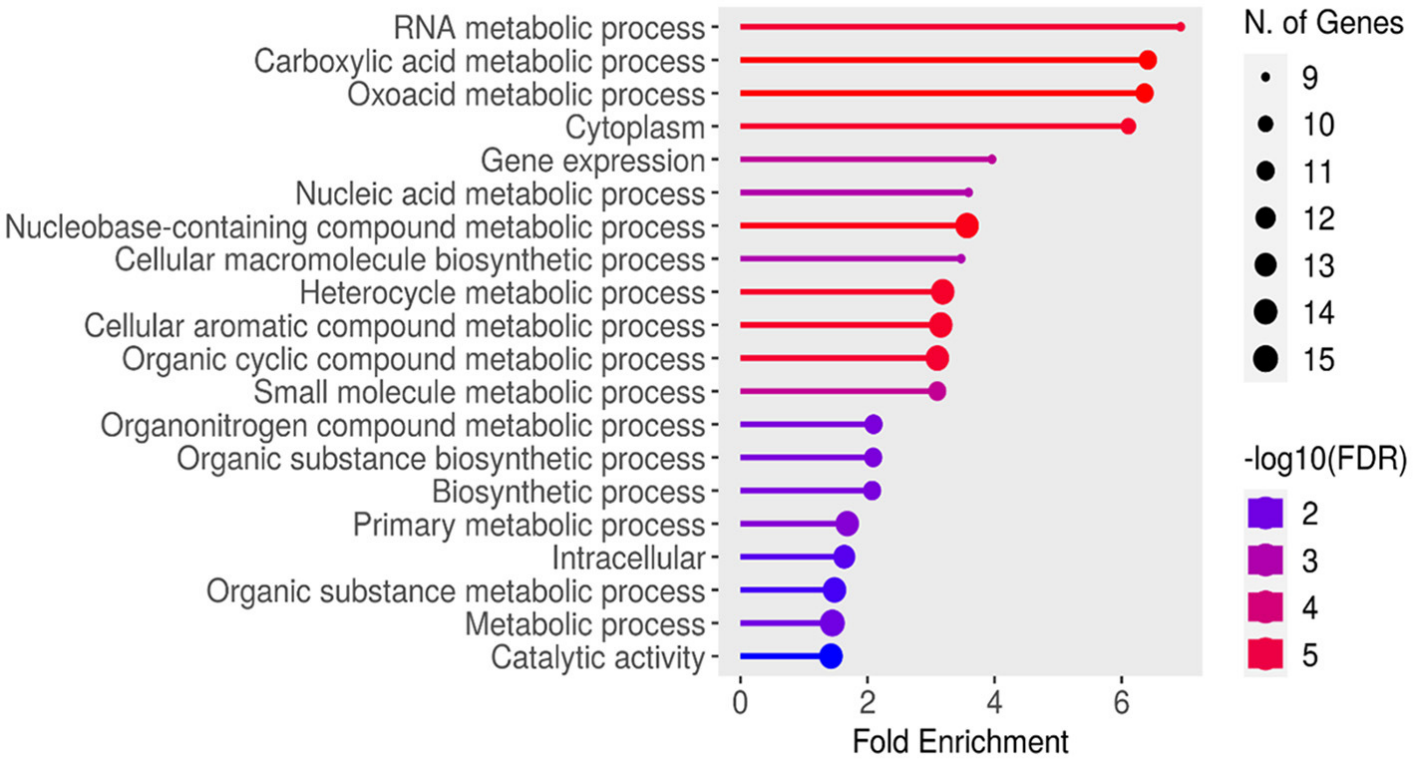
Functional enrichment analysis using ShinyGO ontology.

Figure 4 displays the results of an enrichment analysis of various BP, MF, and CC with their statistical significance and the number of associated genes highlighted. The *X*-axis is used to indicate fold enrichment, reflecting the frequency of each BP in the dataset compared to what would be expected by chance. The fold enrichment value is represented by the length of each bar, and BP are depicted on the *Y*-axis. The colors of the bars, ranging from blue to red, represent –log10 (FDR) values, with red hues indicating higher statistical significance. The number of genes linked to each process is indicated by the size of the circles at the end of the bars. High fold enrichment and significance are observed for processes such as “RNA metabolic process,” “Carboxylic acid metabolic process,” and “Oxoadic acid metabolic process,” as indicated by long red bars with large circles. In contrast, lower fold enrichment and significance are exhibited by processes like “Catalytic activity” and “Metabolic process,” represented by shorter blue bars with smaller circles. A detailed summary of the BP most prominently represented in the dataset is provided by this visual depiction, which aids in understanding gene functions and their interactions.

**Figure 5:**
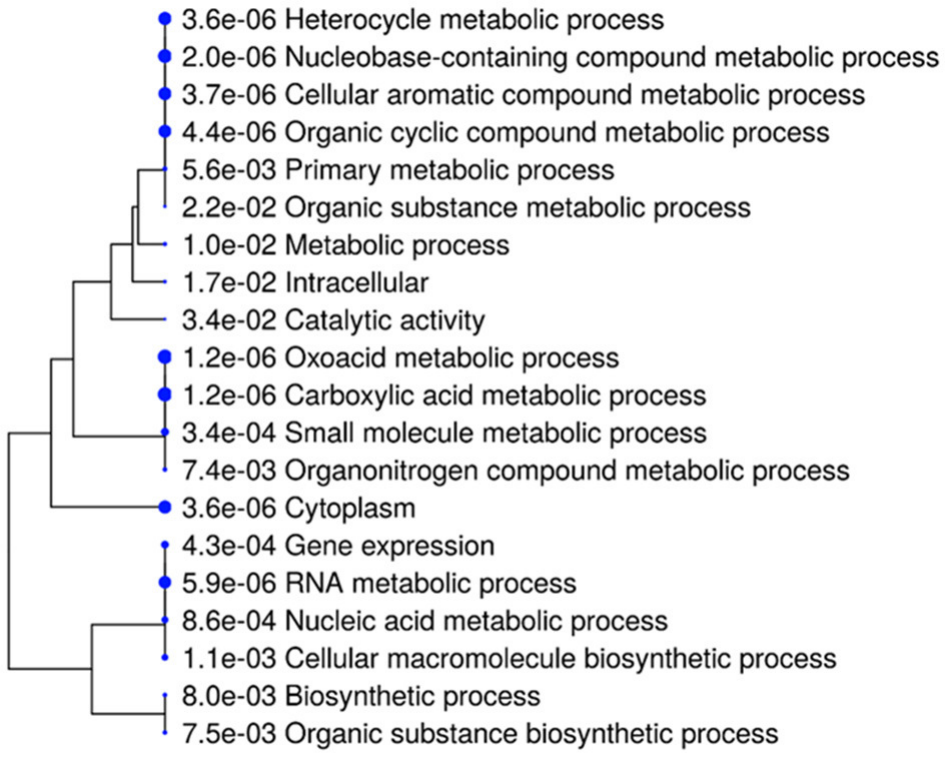
A hierarchical clustering tree illustrating the correlation among significant pathways of the top 20 genes was generated in ShinyGO. Pathways with numerous shared genes were clustered, with larger dots representing more significant *p*-values.

The hierarchical clustering dendrogram is used to visualize relationships among BP, MF, and CC identified through gene enrichment analysis (Figure 5). Each process is represented by a node marked with a blue circle and labeled accordingly. The degree of relatedness is indicated by branch lengths, with shorter branches representing closer associations. Statistical significance is denoted by *p*-values in scientific notation adjacent to each node (Ramesh Babu, 2023). A *p*-value of 3.6 × 10^−6^ for the “Heterocycle metabolic process” indicates strong enrichment. Closely related processes, such as “Heterocycle metabolic process,” “Nucleobase-containing compound metabolic process,” and “Cellular aromatic compound metabolic process,” are grouped on shorter branches, while less related processes, like “Catalytic activity” and “Metabolic process,” are connected by longer branches. The use of this dendrogram is significant as enriched processes are identified and highlighted, gene functions and interactions are clarified, and hypotheses are generated. It also supports the discovery of novel biological connections and guides research toward potential therapeutic targets.

**Figure 6:**
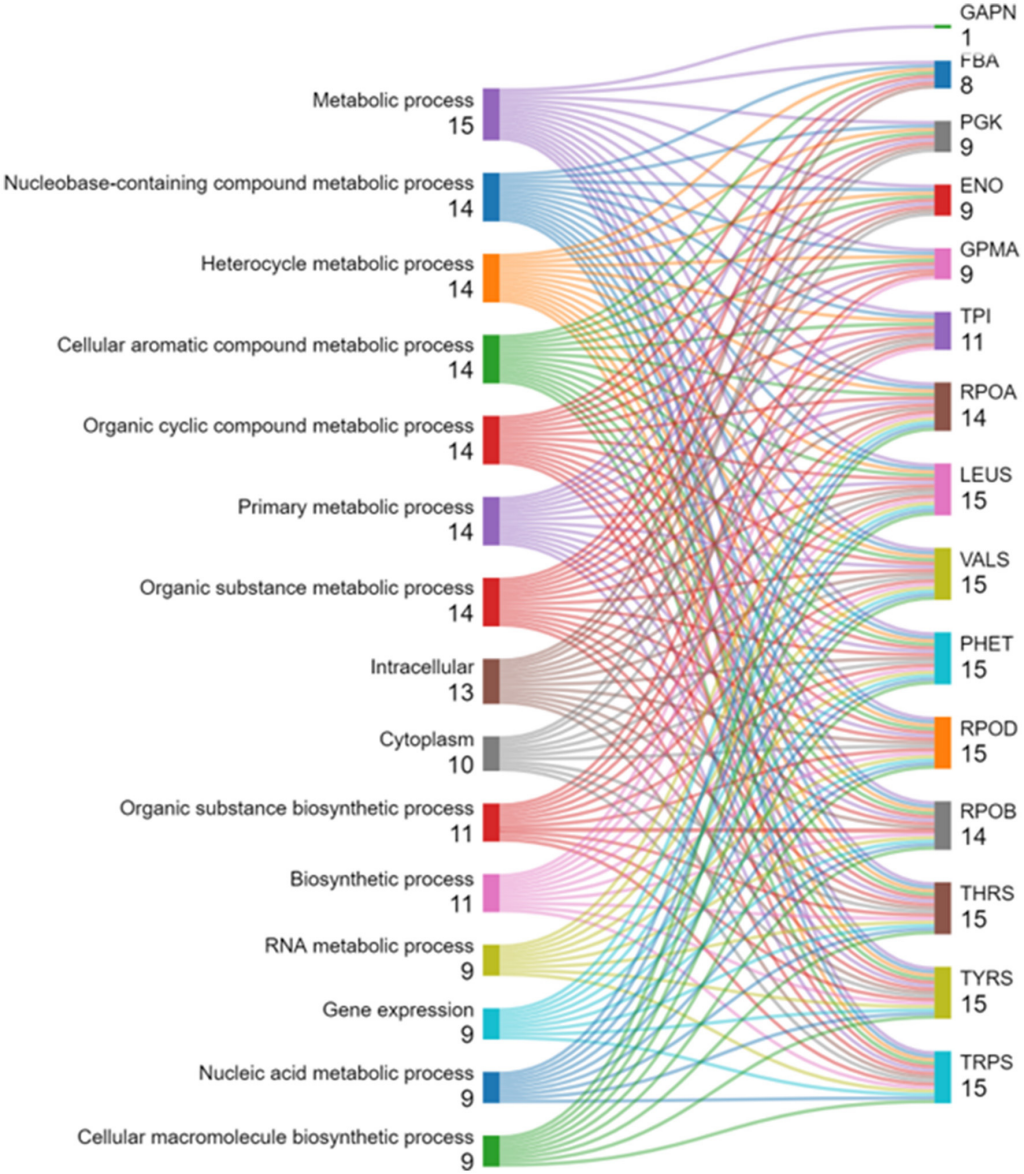
Pathway analysis using ShinyGO 0.80.

Pathway analysis was conducted on the top 20 hub genes using ShinyGO (version 0.80; Zhuang et al., 2022). An overview of the most enriched pathways and their associated genes is presented in Figure 6, which displays the enriched pathways and their associated genes from a given gene set. Pathways are ranked by the number of genes involved, with those having the highest counts emphasized. Most input genes are linked to metabolic pathways, including nucleobase, heterocycle, aromatic compound, and organic substance metabolism, each involving about 14–15 genes. Biosynthetic pathways, such as organic substance biosynthesis and general biosynthesis, are highlighted with ~11 genes. Cellular localization pathways are also shown, with 13 genes related to intracellular processes and 10 to the cytoplasm. The Figure details specific genes in these pathways, providing insights into their roles and relationships within the biological context.

### 3.11 Homology modeling of the identified target

In *S. pneumoniae*, the RNA polymerase sigma factor SigA (rpoD) is crucial for transcription, yet its crystal structure is not present in the Protein Data Bank (PDB). Despite the availability of an AlphaFold-predicted structure on UniProt, a homology model was constructed using Swiss Model (Supplementary Figure 1) to facilitate additional analysis and validation (Rajkhowa et al., 2017). From the UniProtKB database, the primary sequence of SigA, which comprises 369 amino acids, was retrieved (sequence ID: POA4I9, entry WP_000201898.1). The modeling was focused on the sigma-70 factor domain-2, specifically targeting amino acid residues from M1 to I206. This approach was informed by literature and bioinformatics tools, which identified several domains in rpoD, but domain 2 (Region-2; Guo et al., 2018; Lonetto et al., 1992) is defined as conserved binding site so we have considered domain 2 but as the stretch is very small so we have considered from residue no 1–206 to design our model. Structural insights into the conserved domains of the sigma factor, particularly the binding site, are offered by the homology model, which is considered essential for understanding its interactions within the transcription machinery.

### 3.12 Modeled structure validation

In this study, the structural models of the target protein were optimized using the GALAXY refinement tool (Ko et al., 2012), leading to notable improvements across several evaluation metrics. The initial model showed a RMSD (Root Mean Square Deviation) of 0 Å, but refinement quality was lacking. Following the refinement, the lowest RMSD of 0.708 Å was achieved by MODEL 4, indicating a closer alignment with the reference structure. The MolProbity score improved from **0.984** in the initial model to **0.755** in MODEL 4, reflecting enhanced stereochemical quality. The clash score decreased from 1.2 to 0.8, indicating a reduction in steric clashes, and the number of poor rotamers was eliminated, demonstrating optimal side-chain conformations in MODEL 4. The proportion of residues situated in favorable regions of the Ramachandran plot was elevated to **99.5%**, and the GALAXY energy decreased to −5794.77, indicating enhanced stability. Therefore, **MODEL 4** was identified as the most refined and accurate structure, making it the preferred candidate for further studies, as shown in Table 4.

**Table 4:** Selection of the optimal model after refinement.

**Model**	**RMSD**	**MolProbity**	**Clash score**	**Poor rotamers**	**Rama favored**	**GALAXY energy**
Initial	0.000	0.984	1.2	1.2	97.5	−3433.22
MODEL 1	0.853	0.814	1.1	0.6	99.5	−5813.14
MODEL 2	1.502	0.866	1.4	0.0	99.5	−5807.18
MODEL 3	0.829	0.755	0.8	0.6	99.0	−5799.21
**MODEL 4**	**0.708**	**0.755**	**0.8**	**0.0**	**99.5**	**−5794.77**
MODEL 5	0.846	0.755	0.8	0.0	99.5	−5787.65
MODEL 6	0.739	0.814	1.1	0.0	99.5	−5787.58
MODEL 7	0.800	0.866	1.4	0.6	99.0	−5783.87
MODEL 8	0.736	0.814	1.1	0.6	99.5	−5783.26
MODEL 9	0.881	0.755	0.8	0.0	99.5	−5782.61
MODEL 10	0.862	0.866	1.4	0.0	99.5	−5782.48

Values of MODEL 4 has been made bold to show the best values as compared to the other models.

The structure verification process is described in the subsequent sections, following the employment of various tools for model validation.

### 3.13 Protein confirmation using PSIPRED

**Figure 7:**
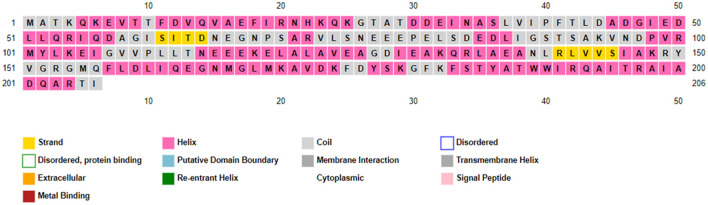
This sequence plot provides a comprehensive overview of the secondary structure elements and functional annotations of the protein, which is valuable for both structural biology studies and functional analysis.

Figure 7 shows that a higher prevalence of alpha helices compared to beta sheets was indicated in the RNA polymerase sigma factor SigA by the PSIPRED analysis (Ashraf et al., 2022). Further validation of the predicted secondary structural elements, including the formation of alpha helices and beta sheets, was performed through modeling with the Modeler tool.

### 3.14 Validation of the modeled protein using PROCHECK, ERRAT, and verify 3D

Protein models were assessed using ERRAT, Verify 3D, and Ramachandran plot analyses (Ashraf et al., 2022). The evaluation identified **MODEL 4** as the most optimal. ERRAT scores were consistently close to 100, reflecting minimal errors in atomic interactions and confirming high structural reliability. In the Verify 3D analysis, compatibility percentages were observed to range from 67.48% to 100%, with **MODEL 4** achieving full compatibility at 100%, demonstrating complete consistency between its three-dimensional structure and one-dimensional sequence. According to the Ramachandran plot analysis, the percentage of residues in favored regions was found to range from 67.48% to 100%, with ~3%−4% located in allowed regions and few or no residues identified as outliers. Notably, 100% of residues in **MODEL 4** were found in favored regions, suggesting that all residues adopted energetically favorable conformations, indicative of a highly refined structure. Collectively, these metrics confirm that the refined models, particularly **MODEL 4**, exhibit high quality and structural integrity, rendering them suitable for further analysis and potential experimental validation, as presented in Table 5.

**Table 5:** PROCHECK, ERRAT, and verify 3D analysis.

**Model_no**.	**ERRAT**	**Verify 3D (%)**	**3D−1D**	**Ramachandran plot**		
				**Favored (%)**	**Allowed (%)**	**Outliers (%)**
MODEL 1	100	70.39	>=0.1	96.80	3.20	0.00
MODEL 2	100	67.48	>=0.1	95.70	3.70	0.00
MODEL 3	99.49	70.39	>=0.1	96.30	3.20	0.50
MODEL 4	100	70.87	>=0.1	96.80	3.20	0.00
MODEL 5	100	69.90	>=0.1	95.70	4.30	0.00
MODEL 6	100	70.39	>=0.1	96.30	3.20	0.00
MODEL 7	100	69.42	>=0.1	96.30	3.20	0.50
MODEL 8	100	70.39	>=0.1	96.30	3.20	0.00
MODEL 9	100	70.39	>=0.1	96.30	3.20	0.50
MODEL 10	100	68.93	>=0.1	96.80	3.20	0.00

### 3.15 Active site identification

The protein analysis identified key domains, including the Sigma-70 factor domain-2 (residues 135–205), a conserved binding site, and additional domains involved in transcriptional regulation and DNA binding. Annotations on domain structures and functional sites were obtained from UniProt, and motif search tools identified conserved sequences and motifs, allowing precise characterization of the protein's interactive regions and regulatory elements, as detailed in Supplementary Tables 1A, B.

Domain 2 of the sigma factor σA was found to be essential for transcription initiation (Guo et al., 2018; Lonetto et al., 1992). Specific promoter sequences, particularly the −10 region (Pribnow box), are recognized and bound by this domain, facilitating the formation of the RNA polymerase-promoter complex. The transcription complex is stabilized by Domain 2, ensuring that RNA polymerase remains bound to the promoter. The transition from the closed to the open complex, allowing DNA strand unwinding for RNA synthesis, is promoted by this domain. Interactions with regulatory proteins are mediated by Domain 2, influencing transcriptional responses to environmental changes and regulating gene expression.

**Figure 8:**
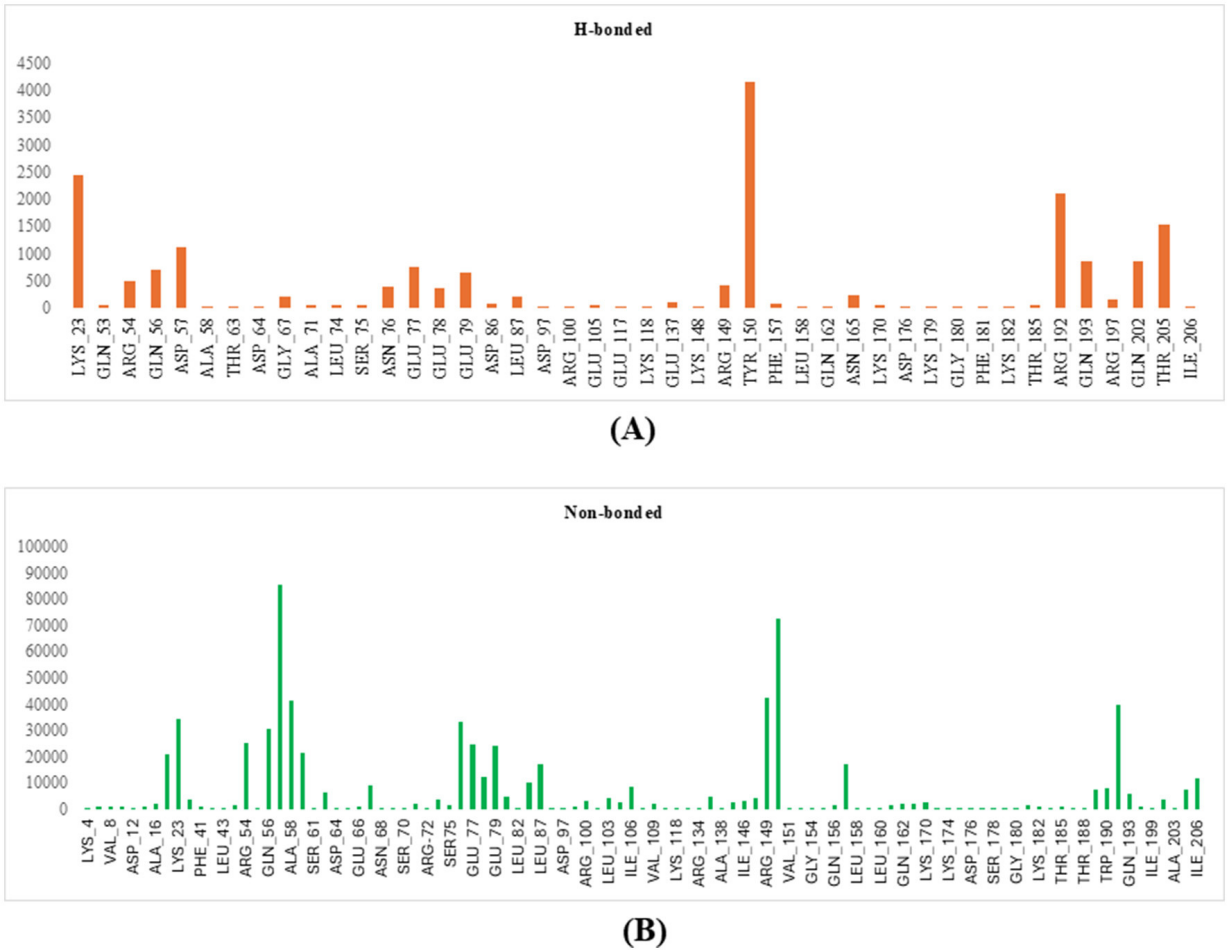
Figure showing the key amino acid residues in the modeled protein using FTMap **(A)** H-bonds; **(B)** non-bonded interactions.

Using the FTMap server, key amino acid residues in the protein involved in both hydrogen-bonded and non-bonded interactions with the ligand were identified, as shown in Figures 8A, B (Pagare et al., 2021). Residues involved in high-frequency hydrogen-bonded interactions include LYS_23, ARG_54, GLN_56, ASP_57, GLY_67, ASN_76, GLU_77, GLU_78, GLU_79, ARG_149, TYR_150, GLN_193, GLN_202, and THR_205, which are crucial for stabilizing the ligand-protein complex and determining binding specificity. Non-bonded interactions, including hydrophobic contacts and van der Waals forces, are mediated by residues such as ALA_16, ILE_19, ARG_54, GLN_56, ASP_57, ALA_58, GLY_59, ASN_76, GLU_77, GLU_78, GLU_79, ASP_86, LEU_87, ARG_149, TYR_150, PHE_157, TRP_189, TRP_190, ARG_192, THR_205, and ILE_206. These residues are identified as significant contact points that contribute to the enhancement of binding affinity. Interactions of moderate and low frequency also contribute to the overall stability of the binding. Valuable insights into the binding mechanism are provided by this detailed analysis, which also serves as a foundation for designing molecules that target these specific interactions.

To elucidate the surface topology and functional regions of the protein structure, the CASTp (Computed Atlas of Surface Topography of Proteins) tool was utilized for analysis. A three-dimensional representation of the protein is provided in Supplementary Figure 2, with the backbone displayed in a ribbon format, and various surface pockets are highlighted by colored spheres. Different active sites are marked by these spheres, which identify key binding pockets essential for the protein's function. Four distinct pockets were identified, each characterized by its solvent-accessible surface area (SA) and volume, as shown in Supplementary Figure 2 and Supplementary Table 3. The measurements of solvent-accessible surface area indicate that **Pocket 1** has the largest area and volume, suggesting a prominent role in ligand binding or enzymatic activity, while **Pocket 4**, despite being smaller, may still have functional significance.

### 3.16 Molecular dynamic simulation of the native protein

**Figure 9:**
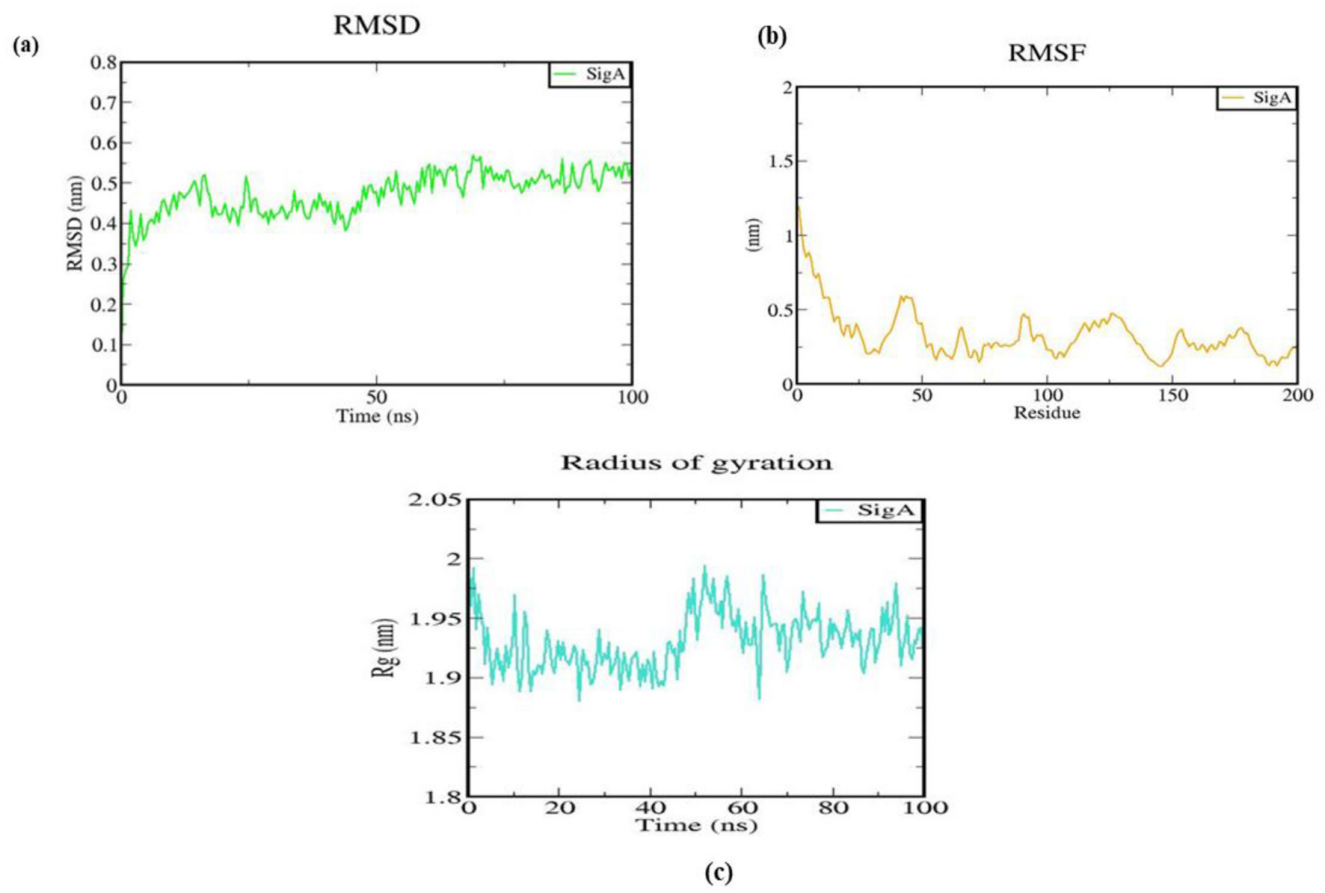
Structural behavior and density of the native protein. **(A)** RMSD plot; **(B)** RMS fluctuation plot; and **(C)** Radius of gyration with time evolution plot.

All-atom MD simulations were conducted on the native SigA protein model for 100 ns to evaluate its structural changes and dynamics using root-mean-square deviation (RMSD) analysis (Jairajpuri et al., 2021; Rajkhowa et al., 2017, 2022). Insights into the structural stability of the SigA protein over time were provided by the RMSD graph. An initial rapid increase in RMSD from 0 to ~0.4 nm indicated significant conformational changes as the protein underwent equilibration. Following this phase, the RMSD values fluctuated between 0.4 and 0.6 nm, as shown in Figure 9A, suggesting that various conformations were explored while the protein remained relatively stable. After 50 ns, a mean RMSD value was stabilized, with no significant upward or downward trends detected, indicating that an equilibrium state was reached and a stable conformation was maintained for the remainder of the simulation. The final RMSD values, ranging from 0.5 to 0.6 nm, were observed to indicate that substantial deviation from the reference structure was not present after the initial equilibration phase. This stability suggests that the functional conformation of the SigA protein was preserved under the simulated conditions, which is essential for its biological role in *S. pneumoniae*. Understanding the structural stability of SigA helps provide insights into its potential interactions with other molecules and its overall function in the bacterial cell.

Insights into the structural dynamics of the SigA protein were provided by the root mean square fluctuation (RMSF) analysis, with values ranging from ~0.5 to 1.5 nm observed across different residues. Moderate fluctuations (0.5–1.0 nm) were observed in the first 50 residues, indicating relative stability with some flexibility. Higher fluctuations, peaking at around 1.5 nm, were seen in residues 50–100, suggesting increased dynamics likely associated with flexible loops or functional sites. In contrast, residues 100–150 exhibited lower RMSF values (0.5–1.0 nm), indicating greater stability, while residues 150–200 showed slight increases (around 1.0 nm), suggesting some flexibility that may be relevant for interactions, as illustrated in Figure 9B. Overall, these results suggest that while most of SigA maintains a stable structure, certain regions exhibit significant dynamics, which may be important for its biological function and interactions.

The radius of gyration (Rg) plot illustrated how the protein's structure evolved during the 100 ns simulation period. Rg values fluctuated between ~1.8 and 2.05 nm, with most values stabilizing around 1.95 nm, as presented in Figure 9C. These fluctuations indicate that the protein underwent conformational changes, reflecting its dynamic nature. Compact structure retention, essential for the protein's biological activity, was indicated by the stable Rg values. Overall, the plot demonstrates a balance between flexibility and stability in the protein's conformation, indicating that although some degree of motion was present, a predominantly compact and functional structure was retained throughout the simulation.

### 3.17 Virtual screening of the ligands

To determine safety and non-carcinogenic potential, compounds from the DrugBank database were evaluated based on their ADMET (absorption, distribution, metabolism, excretion, and toxicity) characteristics and their carcinogenicity profile. Detailed ADMET profiles and toxicity predictions for these compounds are provided in Supplementary Table 4. Out of 2,509 compounds retrieved, 2,328 were eliminated based on toxicity predictions. Additionally, 1,303 compounds were selected according to hydrophilicity criteria, as detailed in Supplementary Table 5. The protein was determined to be hydrophilic, with a hydropathy index of −0.307; thus, compounds with higher lipophilicity were excluded, while those with hydrophilicity values ranging from +3 to −2 were considered for further analysis.

**Figure 10:**
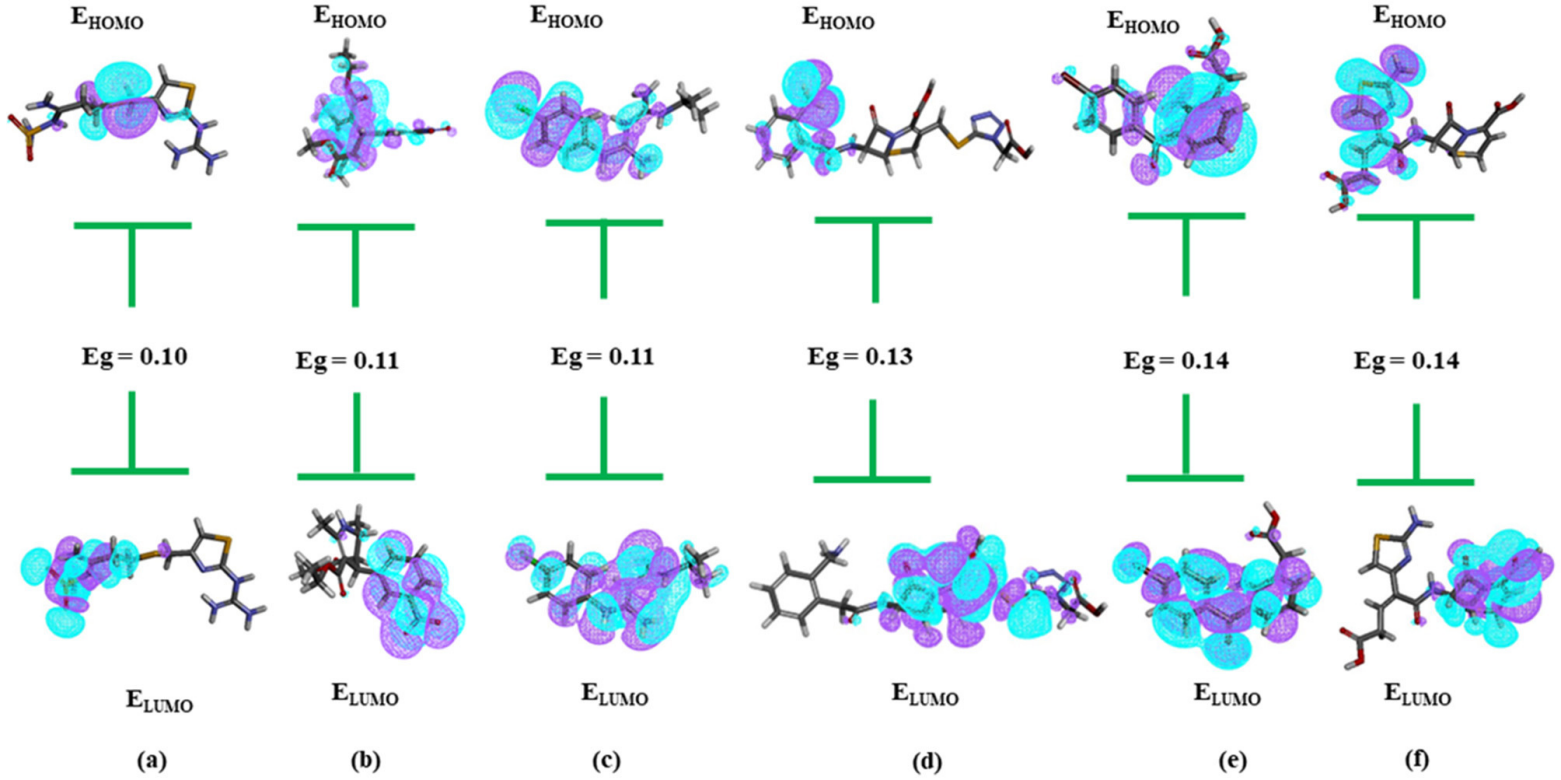
Showing HOMO-LUMO of the six compounds with lowest Eg. **(A)** Famotidine; **(B)** Nitrendipine; **(C)** Proguanil; **(D)** Ceforanide; **(E)** Bromfenac; **(F)** Ceftibuten.

LibDock (Alam and Khan, 2018) molecular docking was applied to refine the selection process, resulting in the identification of the 471 top-scoring compounds, detailed in Supplementary Table 6. Density Functional Theory (DFT) analysis was performed on these compounds, revealing that 22 had negative binding energies, as determined using the Discovery Studio module. Six compounds—**Famotidine, Nitrendipine, Proguanil, Ceforanide, Bromfenac**, and **Ceftibuten**-were identified through further DFT analysis as having the smallest HOMO-LUMO energy gaps, as presented in Supplementary Table 7 and Figures 10A–F.

**Figure 11:**
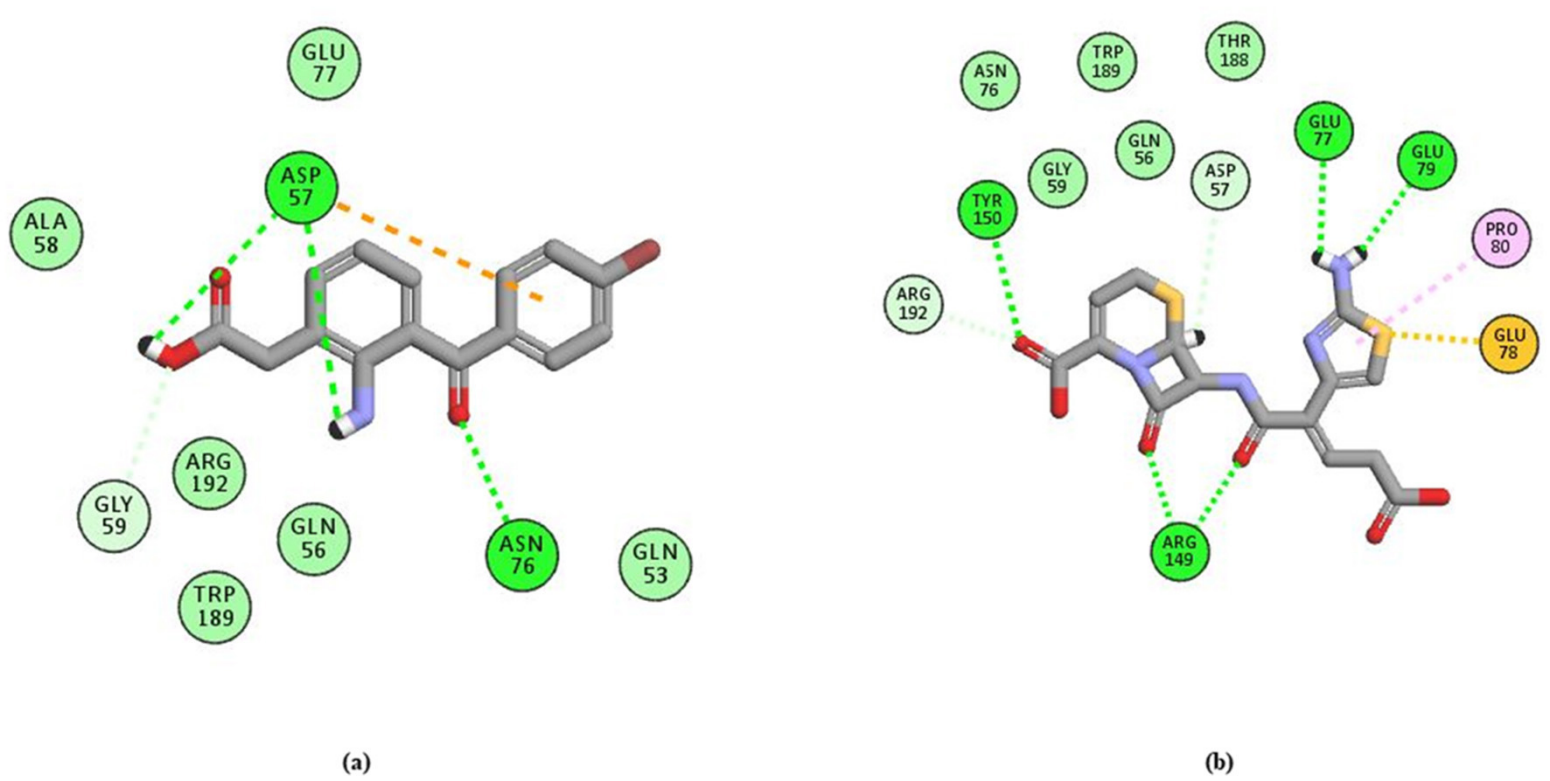
H-bond interaction of **(A)** Bromfenac and **(B)** Ceftibuten with the receptor.

Following the evaluation of hydrogen bond interactions, as detailed in Figure 11 and Supplementary Table 8, Bromfenac (Lee et al., 2023; Ye et al., 2020) and Ceftibuten (Karlowsky et al., 2022; Wiseman and Balfour, 1994) were selected for further analysis. Due to the formation of three hydrogen bonds and the presence of unfavorable steric interactions, Famotidine was excluded. Nitrendipine and Proguanil were dismissed because of insufficient experimental evidence. Additionally, Ceforanide was eliminated for forming only a single hydrogen bond.

### 3.18 Molecular dynamics simulation of the protein-ligand complex

**Figure 12:**
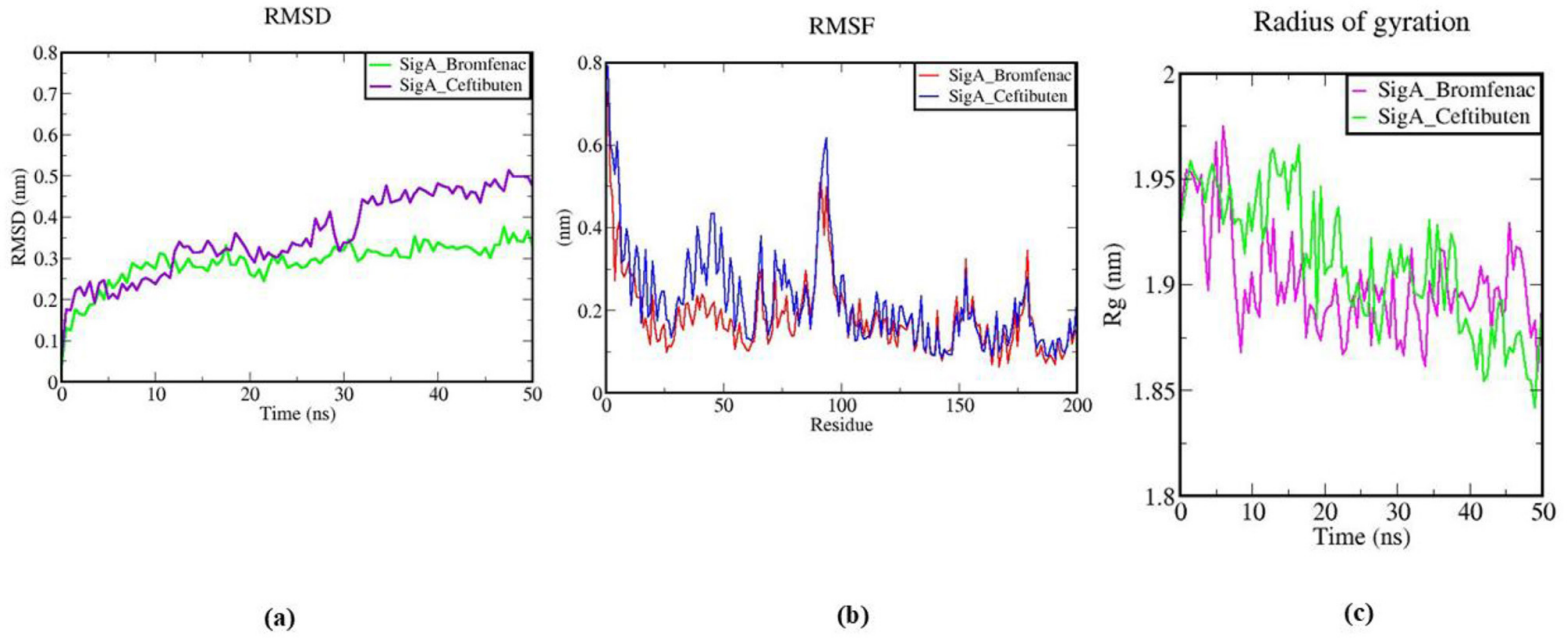
**(A)** RMSD plot of SigA with Bromfenac and Ceftibuten; **(B)** RMS fluctuation plot of SigA with Bromfenac and Ceftibuten; **(C)** Radius of gyration (Rg) plot of SigA with Bromfenac and Ceftibuten.

Bromfenac and Ceftibuten, when bound to the RNA polymerase sigma factor SigA, underwent 50 ns of all-atom MD simulations. To evaluate the stability and dynamics of the SigA-Bromfenac and SigA-Ceftibuten complexes, systematic and structural parameters were computed (Rajkhowa et al., 2022). Stability was assessed using the RMSD plot, with Bromfenac and Ceftibuten depicted by green and purple lines, respectively. Both complexes exhibited an initial rise in RMSD, with SigA-Bromfenac reaching around 0.2 nm and SigA-Ceftibuten reaching ~0.25 nm. During the mid-phase (10–30 ns), stabilization around 0.3 nm was noted for the SigA_Bromfenac complex, while greater fluctuations were exhibited by the SigA_Ceftibuten complex, reaching up to 0.4 nm. In the final phase (30–50 ns), the RMSD of the SigA_Bromfenac complex was maintained at ~0.3–0.4 nm, whereas the SigA_Ceftibuten complex increased to about 0.5 nm. This indicated greater stability for the SigA_Bromfenac complex, suggesting a more stable interaction with SigA compared to Ceftibuten, as shown in Figure 12A.

The residue flexibility within the two SigA complexes, bound to Bromfenac (depicted by the red line) and Ceftibuten (depicted by the blue line), was illustrated using a plot of root-mean-square fluctuation (RMSF). A lower degree of flexibility was noted in the SigA_Bromfenac complex, indicating a more rigid conformation. Increased flexibility was observed around residues 40–60 and 90–110 in both complexes, with higher peaks recorded in the SigA_Ceftibuten complex. Enhanced fluctuations were also noted around residues 120–140 in the SigA_Ceftibuten complex. These results indicated that a more stable structure was maintained by the SigA_Bromfenac complex, reinforcing that Bromfenac interacted more stably with SigA, as depicted in Figure 12B.

During the 50 ns MD simulation, the compactness of the SigA complexes with Bromfenac (indicated by the purple line) and Ceftibuten (indicated by the green line) was demonstrated through a plot of the radius of gyration (Rg). Initial Rg values of ~1.95 nm were recorded for both complexes, suggesting similar compactness. Over time, stabilization around 1.9 nm was observed for the SigA_Bromfenac complex, indicating increased compactness. In contrast, fluctuations between 1.85 and 1.95 nm were exhibited by the SigA_Ceftibuten complex, indicating less consistent compactness. Overall, the findings demonstrated that a more stable and compact structure was maintained by the SigA_Bromfenac complex compared to the SigA_Ceftibuten complex, further supporting that Bromfenac formed a more stable interaction with SigA, as presented in Figure 12C.

### 3.19 Assessment of binding energy

The binding interactions of Bromfenac and Ceftibuten were evaluated using MM-PBSA (Molecular Mechanics Poisson-Boltzmann Surface Area) analysis based on data from MD. The van der Waals energy for Bromfenac was measured at −80.983 ± 20.129 kJ/mol, while Ceftibuten was found to be −99.688 ± 26.800 kJ/mol, indicating favorable interactions with a stronger affinity observed for Ceftibuten. The electrostatic energy values were recorded as −75.863 ± 40.066 kJ/mol for Bromfenac and −199.464 ± 43.954 kJ/mol for Ceftibuten, suggesting attractive interactions, with significantly stronger electrostatic interactions attributed to Ceftibuten.

The polar solvation energy for Bromfenac was determined to be 141.547 ± 54.370 kJ/mol, whereas Ceftibuten exhibited a higher value of 325.643 ± 62.032 kJ/mol, indicating unfavorable solvation effects for Ceftibuten. The solvent-accessible surface area (SASA) values were measured for Ceftibuten −11.036 ± 1.771 kJ/mol for Bromfenac and −15.474 ± 2.672 kJ/mol for Ceftibuten, suggesting favorable interactions regarding surface exposure.

The binding energy for Bromfenac was calculated as −26.335 ± 29.105 kJ/mol, indicating favorable binding, while a positive binding energy of 11.016 ± 26.392 kJ/mol was observed for Ceftibuten, suggesting reduced binding efficacy. Overall, it was concluded that while stronger interaction energies were exhibited by Ceftibuten, **Bromfenac** was identified as better candidate for effective binding, as shown in Table 6. Although antimicrobial activity is not directly exhibited by Bromfenac alone, antibacterial and anti-inflammatory effects are demonstrated when it is combined with nanoparticles. For example, endophthalmitis can be treated by AuAgCu_2_O-bromfenac sodium nanoparticles (Ye et al., 2020; AuAgCu_2_O-BS NPs) through the combination of antibacterial and anti-inflammatory actions. MRSA (methicillin-resistant *Staphylococcus aureus*) is eradicated by these nanoparticles through photodynamic effects and the release of metal ions, with the bacterial membrane being disrupted and cell death being caused. Therefore, the potential of Bromfenac as a repurposed drug against *S. pneumoniae* may be explored experimentally.

**Table 6:** Binding free energy calculation of the protein and ligand complexes.

**Energy**	**SigA_Bromfenac (kJ/mol)**	**SigA_Ceftibuten (kJ/mol)**
van der Waals	−80.983 ± 20.129	−99.688 ± 26.800
Electrostattic	−75.863 ± 40.066	−199.464 ± 43.954
Polar solvation	141.547 ± 54.370	325.643 ± 62.032
SASA	−11.036 ± 1.771	−15.474 ± 2.672
Binding	−26.335 ± 29.105	11.016 ± 26.392

## 4 Discussion

High rates of meningitis, lobar pneumonia, otitis media, and bacteremia are caused by *S. pneumoniae*. The potential increase in pneumococcal infections is suggested by the rising resistance to penicillin and the limited availability of pneumococcal vaccines, highlighting the urgent need for the identification of new therapeutic targets. The subtractive genomics method is employed in bioinformatics to identify pathogen-specific targets. This *in silico* approach involves a thorough examination of the pathogen's genome to identify key proteins involved in the disease, with the host genome excluded from consideration.

Although previous studies have explored subtractive genomics in *S. pneumoniae* (Khan et al., 2022; Sheoran et al., 2022; Wadood et al., 2018), the TIGR4 strain remains highly prevalent. Over the past decade, this specific serotype has been the leading cause of childhood meningitis, pneumonia, and bacteremia in developing countries. Sequencing and publication of the complete genome for the virulent serotype 4 strain TIGR4 were accomplished by The Institute for Genomic Research (TIGR) in 2001. Despite its identification over 20 years ago, the TIGR4 strain still shows significant virulence in murine models (Tettelin et al., 2001) and continues to be a key focus in pneumococcal pathogenesis research. Thus, the current study is directed at identifying new drug targets within the *S. pneumoniae* TIGR4 strain.

Analysis was conducted on 2,027 proteins from the complete proteome of *S. pneumoniae*. To improve computational efficiency and resource management in large datasets, paralogous sequences were identified and removed. Using the CD-HIT tool with a 90% similarity threshold, 11 paralogous sequences were filtered out, leaving a final dataset of 2,016 proteins.

Given that many antibiotics inadvertently target beneficial human gut microbiota alongside pathogens, the exclusion of pathogen proteins homologous to gut flora proteins from the drug target pool could mitigate adverse side effects. The evolutionary relationship with gut flora was assessed by querying the entire genome of *S. pneumoniae* against gastrointestinal flora reference genomes using BLASTp via the mBodyMap Database. Of the 2,087 gut bacterial sequences, 87 showed homology with gut flora, whereas 2,000 were non-homologous. A BLASTp search against the human genome identified 1,981 homologous proteins, leaving 27 proteins (*E*-value ≤ 10^−5^) that did not exhibit similarity to the human genome. Through a BLASTp search against DEG, 21 genes essential for the viability of *S. pneumoniae* (*E*-value ≤ 10^−100^) were identified, suggesting their potential as drug targets.

Genes essential for cellular survival cannot sustain bacterial life if they are disrupted or degraded. Therefore, a strategic approach for bacterial eradication and disease treatment is provided by targeting these vital proteins, which are considered excellent candidates for the development of vaccines and antibiotics. A total of 69 essential proteins were identified, with 48 shared between host and pathogen and 21 specific to host gut bacteria and pathogen. However, it should be noted that therapeutic applications may not be suitable for all essential genes due to their potential involvement in host metabolic pathways.

Subcellular localization prediction provides a cost-effective means to infer protein function and guide therapeutic development. Since proteins may localize in multiple cellular compartments, localization data are critical for the rational design of therapeutic agents. Due to the challenges associated with purifying and studying membrane proteins, drug targets are generally selected from cytoplasmic proteins.

For drug screening purposes, the DrugBank database was queried using BLASTp to compare all essential non-homologous proteins involved in distinct bacterial pathways. This comparison led to the identification of four proteins with potential for drug targeting: RNA polymerase sigma factor SigA, Oligopeptide transport system permease protein AmiD, Fructose-bisphosphate aldolase, and Tryptophan-tRNA ligase. The primary focus was placed on RNA polymerase sigma factor SigA (Große et al., 2022; Martins et al., 2024), while the other proteins were excluded based on the reasons outlined in the manuscript.

The Swiss-Model was utilized to predict the three-dimensional structures of the selected target proteins through a homology modeling approach. For molecular docking analysis, which is essential in drug discovery for predicting interactions between proteins and ligands, compounds from DrugBank (FDA-approved) were employed. Molecular docking using LibDock identified 471 compounds with the highest docking scores. Density Functional Theory (DFT) analysis further refined this list to 22 compounds with negative binding energies. Of these, six compounds-Bromfenac, Ceftibuten, Famotidine, Nitrendipine, Proguanil, and Ceforanide- exhibited the smallest HOMO-LUMO energy gaps. Bromfenac and Ceftibuten were prioritized for further analysis based on favorable hydrogen bonding interactions. Famotidine was excluded due to unfavorable steric interactions, Nitrendipine and Proguanil were removed due to insufficient experimental evidence, and Ceforanide was excluded due to limited hydrogen bonding (one bond). The stability and dynamics of these interactions over time were assessed through MD simulations.

For ensuring effective metabolism and therapeutic efficacy, the absorption and distribution of an ideal drug candidate throughout the body should be readily achieved. High costs can be incurred when drug candidates are rejected during clinical trials due to adverse side effects. Therefore, ADMET (absorption, distribution, metabolism, excretion, and toxicity) profiling, an essential phase in drug discovery, was carried out using Discovery Studio, which showed favorable pharmacokinetic properties for specific compounds. Of 2,509 compounds screened, 2,328 were excluded based on toxicity. Compounds with hydrophilicity values between +3 and −2 were retained for further analysis, given the hydrophilic nature of the target protein (hydropathy index −0.307).

The MM-PBSA method was used to calculate the binding free energies of the docked complexes, offering quantitative assessments of interaction strength. More stable and compact binding with RNA polymerase sigma factor SigA was demonstrated by **Bromfenac** compared to Ceftibuten among the compounds tested, indicating its potential as a promising drug candidate.

The time and cost associated with drug development can be significantly reduced through drug repurposing. In this study, existing drugs were screened for interactions with identified targets, and Bromfenac, a non-steroidal anti-inflammatory drug, was recognized as a potential candidate for repurposing against *S. pneumoniae*. Although no reports have indicated antimicrobial activity for Bromfenac, some studies propose that it possesses antibacterial and anti-inflammatory properties, supported by experimental evidence. While Bromfenac does not exhibit direct antibacterial action, antibacterial and anti-inflammatory effects relevant to our research are observed when it is combined with nanoparticles.

For instance, AuAgCu_2_O-bromfenac sodium nanoparticles (AuAgCu_2_O-BS NPs; Ye et al., 2020) are utilized to combine antibacterial and anti-inflammatory effects for the treatment of endophthalmitis after cataract surgery. The ability to eradicate methicillin-resistant *Staphylococcus aureus* (MRSA) is attributed to these nanoparticles due to their photodynamic properties and the release of metal ions. It should be noted that the antibacterial effect of AuAgCu_2_O NPs is not directly influenced by Bromfenac sodium alone. However, a bactericidal function is demonstrated by AuAgCu_2_O-BS NPs through the disruption of the bacterial membrane, leading to its shrinkage. Interaction with the cell membrane occurs via the nanoparticles, which may affect its permeability, while metal ions are released that penetrate the bacteria.

Significant antibacterial effects in the treatment of endophthalmitis have been observed in studies involving AuAgCu_2_O-BS NPs used alongside laser treatment. Investigating the efficacy of Bromfenac against *S. pneumoniae* would be worthwhile. Therefore, it can be suggested that Bromfenac might serve as a potentially effective repurposed drug against *S. pneumoniae*.

The identification of key hub genes and their associated pathways offers significant insights into the molecular biology of *S. pneumoniae* and establishes a foundation for novel therapeutic strategies. The structural integrity and reliability of the refined protein models further support their potential for experimental validation. The speed of discovering new drug targets for drug-resistant pathogens is enhanced by the use of bioinformatics tools, as demonstrated by the results of this study. The potential for addressing bacterial infections through both new drug development and drug repurposing is emphasized.

## 5 Conclusion

The drug discovery process has been significantly advanced through the utilization of genome and proteome sequence analysis of various pathogens with bioinformatics tools. The use of *in silico* methods has been prompted by the increasing incidence of drug resistance to identify therapeutic targets that are not homologous with the host proteome. To enhance this process, a subtractive genomics approach has been employed to identify essential proteins that are non-homologous and not part of gut flora, which can be explored for developing new therapeutic agents against *S. pneumoniae*. The targeting of strain-specific, non-homologous essential bacterial proteins is anticipated to facilitate disease eradication while minimizing adverse effects on the host. The impact of these targets on the survival and pathogenicity of *S. pneumoniae* is required to be evaluated through additional *in vivo* and *in vitro* studies.

Despite its many benefits, this approach has limitations due to the predictive nature of the tools used. The tools are based on algorithms, and errors may occur, as these algorithms are designed and coded by humans and may have limited specificity for certain tasks.

## Data Availability

The original contributions presented in the study are included in the article/Supplementary material, further inquiries can be directed to the corresponding authors.
