# Supplementary material for: Subtractive genomics and drug repurposing strategies for targeting Streptococcus pneumoniae: insights from molecular docking and dynamics simulations
Source: Front Microbiol. 2025 Mar 18;16:1534659. doi: 10.3389/fmicb.2025.1534659 (PMC11958985; doi:10.3389/fmicb.2025.1534659)
Supplement: Supplementary file 1 [file Data_Sheet_1.docx]

**Subtractive Genomics and Drug Repurposing Strategies for Targeting *Streptococcus pneumoniae*: Insights from Molecular Docking and Dynamics Simulations**

Borakha Bura Gohain,^1^ Bhaskar Mazumder,^2^ Sanchaita Rajkhowa,^1*^ Sami A. Al-Hussain,^3^ Magdi E. A. Zaki,^3*^

^1^Centre for Biotechnology and Bioinformatics, Dibrugarh University, Dibrugarh 786004, Assam, India

^2^Department of Pharmaceutical Sciences, Dibrugarh University, Dibrugarh 786004, Assam, India

^3^Department of Chemistry, Imam Mohammad Ibn Saud Islamic University (IMSIU), Riyadh, Saudia Arabia.

*Corresponding author (s): (Dr. Sanchaita Rajkhowa) [s_rajkhowa@dibru.ac.in](mailto:s_rajkhowa@dibru.ac.in) (ORCID ID: 0000-0002-4834-2654) and (Dr. Magdi E. A. Zaki) [mezaki@imamu.edu.sa](mailto:mezaki@imamu.edu.sa)

**SI Table 1(A): Domains Found based on Uniport**

| Type | ID_position | Description |
| --- | --- | --- |
| Region | 63-85 | Disordered |
| Region | 135-205 | Sigma-70 factor domain-2 |
| Motif 159-162 | 159-162 | Interaction with polymerase core subunit RpoC |
| Region | 214-290 | Sigma-70 factor domain-3 |
| Region | 303-356 | Sigma-70 factor domain-4 |

**SI Table 1(B) Domain found using Motif Search Tool**

| Pfam | Position (Independent E-value) | Description |
| --- | --- | --- |
| Sigma 70_r3 | 214-290 | Sigma 70 region3 |
| Sigma 70-r2 | 135-205 | Sigma 70 region2 |
| Sigma 70-r4 | 304-356 | Sigma 70 region4 |
| Sigma 70_r1_2 | 97-128 | Sigma 70 region1.2 |
| Sigma 70_r1_1 | 14-85 | Sigma 70 region1.1 |
| DBD_HTH | 296-311 | Putative DNA-binding domain |
| DUF23I6 | 235-285 | Uncharacterized protein conserved in bacteria |
| DprA_WH | 221-254 | DprA winged helix domain |


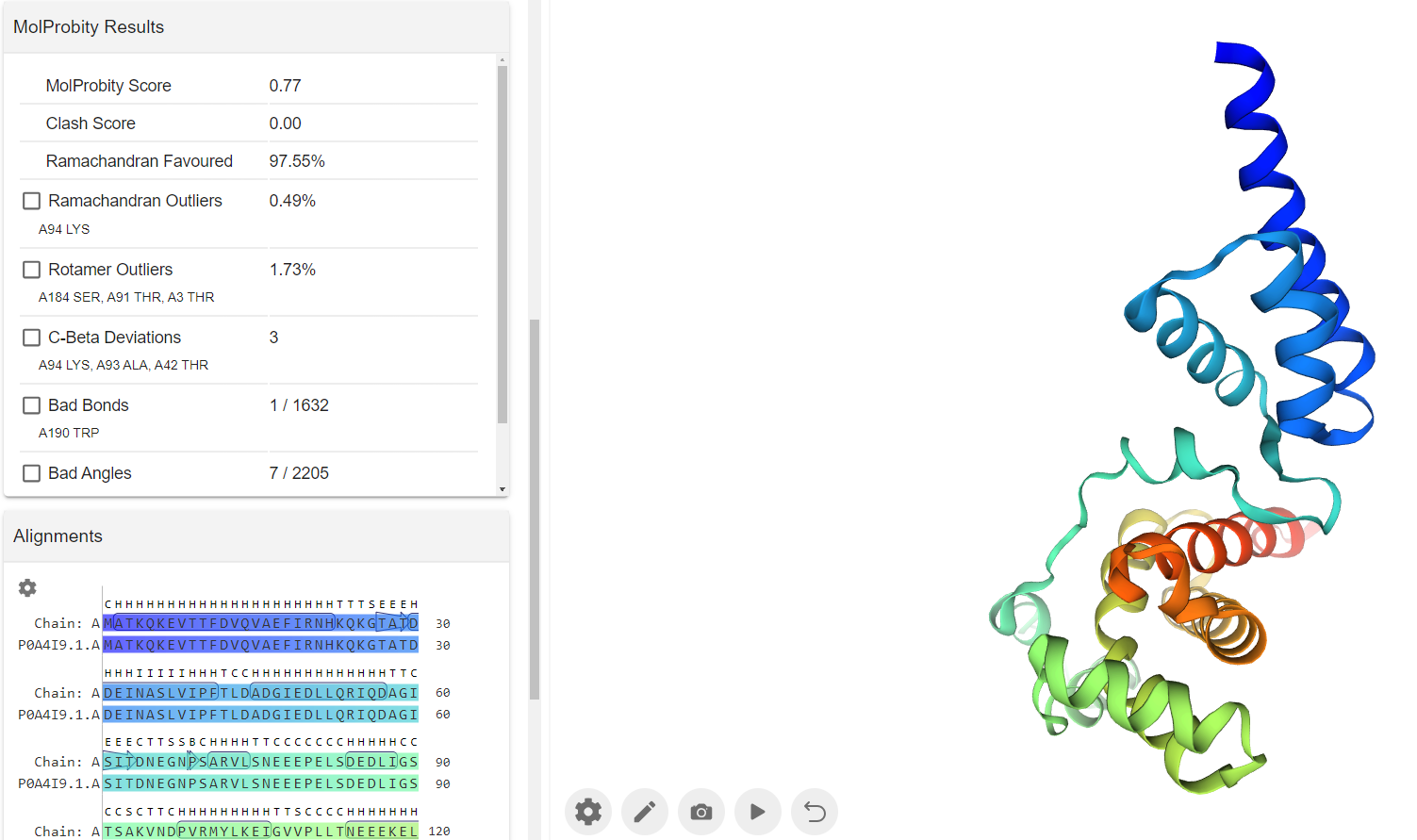


**Figure 1:** Modeled structure of the identified target protein using the Swiss model.

**
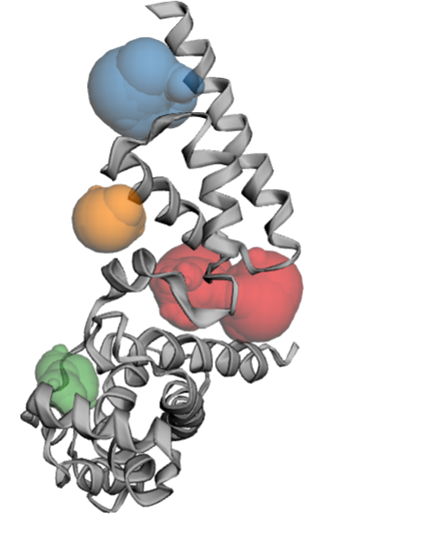
**

**Figure 2:** Characteristics of Identified Pockets

| By Avg_rank (FDR & fold enrichment) | | | | By FDR & fold enrichment | | | | By only FDR | | | | | By only fold enrichment | | | | | | By only genes | | | | | |
| --- | --- | --- | --- | --- | --- | --- | --- | --- | --- | --- | --- | --- | --- | --- | --- | --- | --- | --- | --- | --- | --- | --- | --- | --- |
| All pathways | | **Top pathways** | | **All pathways** | | **Top pathways** | | **All pathways** | | **Top pathways** | | | **All pathways** | | | **Top pathways** | | | **All pathways** | | | **Top pathways** | | |
| Total | Sorted by rpoD | Total | Sorted by rpoD | Total | Sorted by rpoD | Total | Sorted by rpoD | Total | Sorted by rpoD | Total | Sorted by rpoD | Total | | Sorted by rpoD | Total | | Sorted by rpoD | Total | | Sorted by rpoD | Total | | Sorted by rpoD |  |
| 211 | 41 | 19 | 1 | 211 | 41 | 19 | 2 | 211 | 41 | 20 | 2 | 211 | | 41 | 20 | | 2 | 211 | | 41 | 20 | | 15 |  |

**SI Table 2: Analysis Using ShinyGO 0.80**

**SI Table 3: Table showing the Surface Area of the pockets using Castp**

| PocID | Area (SA) (Å²) | Volume (SA) (Å³) |
| --- | --- | --- |
| 1 | 265.517 | 342.27 |
| 2 | 124.027 | 166.593 |
| 3 | 127.402 | 62.408 |
| 4 | 50.673 | 36.495 |

**SI Table 4: ADMET descriptors and Toxicity prediction using Discovery Studios**

**SI Table 5: Selection of the compound based on hydrophilicity**

**SI Table 6: Compounds with the highest LibDock scores**

**SI Table 7: DFT table of the 6 compounds with lowest energy gap (Eg)**

| Sl.No | Name | Optimized Energy | Binding Energy | E_HOMO_ | E_LUMO_ | Eg | IP | EA | Chemical Hardness(η) | Electronegativity (χ) | Chemical Potential (μ) | Electrophilicity Index (ω) | Chemical Softness (Ѕ) |
| --- | --- | --- | --- | --- | --- | --- | --- | --- | --- | --- | --- | --- | --- |
| 1 | Famotidine | -2132.93 | -98.947 | -0.4218 | -0.3199 | 0.10 | 0.4218 | 0.3199 | 0.0510 | 0.3708 | -0.3708 | 0.0035 | 19.6098 |
| 2 | Nitrendipine | -1333.68 | -85.837 | -0.2150 | -0.1006 | 0.11 | 0.2150 | 0.1006 | 0.0572 | 0.1578 | -0.1578 | 0.0007 | 17.4802 |
| 3 | Proguanil | -1220.46 | -64.864 | -0.4635 | -0.3491 | 0.11 | 0.4635 | 0.3491 | 0.0572 | 0.4063 | -0.4063 | 0.0047 | 17.4798 |
| 4 | Ceforanide | -2527.18 | -136.72 | -0.2286 | -0.0946 | 0.13 | 0.2286 | 0.0946 | 0.0670 | 0.1616 | -0.1616 | 0.0009 | 14.9324 |
| 5 | Bromfenac | -3537.7324 | -113.904 | -0.2156 | -0.0758 | 0.14 | 0.2156 | 0.0758 | 0.0699 | 0.1457 | -0.1457 | 0.0007 | 14.2987 |
| 6 | Ceftibuten | -2147.47 | -112.344 | -0.2184 | -0.0746 | 0.14 | 0.2184 | 0.0746 | 0.0719 | 0.1465 | -0.1465 | 0.0008 | 13.9102 |

**SI Table 8: Table 2D interaction between the ligand and receptor**

| **Name** | **Eg** | **H-bonds (lib_dock)** | **Other_bonds (libdock)** | **Non-Bonded(lib_dock)** |
| --- | --- | --- | --- | --- |
| Ceftibuten | 0.14 | **Tyr150, Glu77, Glu79, Arg149** | **Arg192, Glu78,Pro80** | Asn76,Trp189, Thr188, Gly59, Gln56, |
| Famotidine | 0.10 | **Asp57,Arg192,Asn76** | **Glu79** , **Tyr150** | Lys23, Gln56, Gln53, Glu78, Glu77, Arg149, Ile146, Gly59, Ala58 |
| Nitripedine | 0.11 | **Arg192** | **Asp57, Glu 77, Pro80, Glu79, Arg149,Glu79** | Gln53, Gln56, Asn76, Gly59, Trp189, Thr188, Glu78 |
| Bromfenac | 0.14 | **Asp57, Asn76,** | **Gly59** | Ala58, Glu77, Gln53, Arg192, Gln56, Trp189, |
| Proguanil | 0.11 | **Asp44, Thr42** | **Leu51**,**Phe11,Leu43, Arg54** | Asp50, Gly47, Phe41, Glu7, Val8 |
| Ceforanide | 0.13 | **Asn76** | **Gln56** | Asp57, Gln53, arg72, Ser75 |
